# Macrophage-based therapeutic approaches for cardiovascular diseases

**DOI:** 10.1007/s00395-023-01027-9

**Published:** 2024-01-03

**Authors:** Marida Sansonetti, Bashar Al Soodi, Thomas Thum, Mira Jung

**Affiliations:** 1 Institute of Molecular and Translational Therapeutic Strategies (IMTTS), Hannover Medical School, 30625 Hannover, Germany; 2 REBIRTH-Center for Translational Regenerative Medicine, Hannover Medical School, 30625 Hannover, Germany; 3 Fraunhofer Institute for Toxicology and Experimental Medicine (ITEM), 30625 Hannover, Germany

**Keywords:** Macrophages, Immune cells, Inflammation, Gene therapy, Non-coding RNA (ncRNA), Cell therapy, iPSC-derived macrophages, Cardiac remodeling, CVD, Myocardial infarction

## Abstract

Despite the advances in treatment options, cardiovascular disease (CVDs) remains the leading cause of death over the world. Chronic inflammatory response and irreversible fibrosis are the main underlying pathophysiological causes of progression of CVDs. In recent decades, cardiac macrophages have been recognized as main regulatory players in the development of these complex pathophysiological conditions. Numerous approaches aimed at macrophages have been devised, leading to novel prospects for therapeutic interventions. Our review covers the advancements in macrophage-centric treatment plans for various pathologic conditions and examines the potential consequences and obstacles of employing macrophage-targeted techniques in cardiac diseases.

## Introduction

Cardiovascular diseases (CVDs) encompass a spectrum of pathological conditions affecting the heart or blood vessels, including myocardial infarction (MI), arrhythmia and atherosclerosis. Despite significant advancements in research and pharmacological therapies, CVDs such as MI are still a leading cause of global health burden and major contributor to disability [1]. With an aging and expanding global population, the death from CVDs has even raised 60% over the last 30 years and is mostly linked to ischemic heart disease and ischemic stroke-related death (World Heart Federation 2021). According to the global burden of diseases, injuries and risk factors (GBD) study of CVDs related death between 1990 and 2021, metabolic risks, dietary risks and environmental risks are the primary risk factors of CVDs progression and have been found to increase the prevalence of comorbidities like chronic obstructive pulmonary disease (COPD) or diabetes [1, 2]. The most common element of all these risk factors is increased tissue inflammation, which is often negatively associated with survival in patients with CVDs. Intensive investigations in recent decades have illuminated the pivotal role of inflammation in the pathogenesis of CVDs. While acute inflammation is a host-protective response against tissue damage or external stimuli, it must be resolved in a timely manner to maintain homeostasis and facilitate optimal tissue repair [3]. In contrast, unresolved inflammation serves as a catalyst for the progression of chronic inflammation and is associated with an increased risk of heart failure (HF) [4]. Therefore, several strategies to regulate and balance inflammation have been proposed as treatment options for CVDs.

The tissue inflammation is now better appreciated by the dynamic activation status of diverse immune cells including neutrophils, macrophages and T cells in diverse disease contexts. Among them, macrophages are the most abundant immune cells in cardiac tissue, playing a crucial role in the maintenance of homeostasis and cardiac development [5, 6]. Moreover, being a key player to balance inflammation and local microenvironment upon injury, the residence and polarization of macrophages are closely related to HF progression including fibrogenesis or cardiac remodeling [7, 8]. The potential for macrophage-targeted therapies, such as modulating macrophage phenotypes, has garnered significant attention in the CVD field in recent years [9]. Advanced techniques in science and research have revealed the existence of various types of macrophages, each exhibiting distinctive characteristics under both steady-state and pathological conditions. These advancements pave the way for precise targeting of specific macrophage phenotypes and their functions in distinctive pathologies, culminating in more effective and efficient treatments. Our review provides a comprehensive overview of ongoing research and potential therapeutic strategies targeted at macrophages in specific CVDs. We also delve into the challenges of targeting macrophages specific to the heart and explore the application of macrophage-based therapies in clinical trials.

## Macrophages: the shield of hearts against CVDs

The heart is a complex organ composed of a diverse array of cell types, each playing a crucial role in its function. While cardiomyocytes, the contractile cells responsible for the heart's pumping action, have traditionally been the focus of research, recent advances have highlighted the significance of non-myocytes, such as endothelial cells, fibroblasts, and immune cells, in maintaining cardiac health. These diverse cell types form a tightly interconnected network that communicates biochemically and biophysically to ensure proper heart function both under normal conditions and in response to injury [10].

**Fig. 1:**
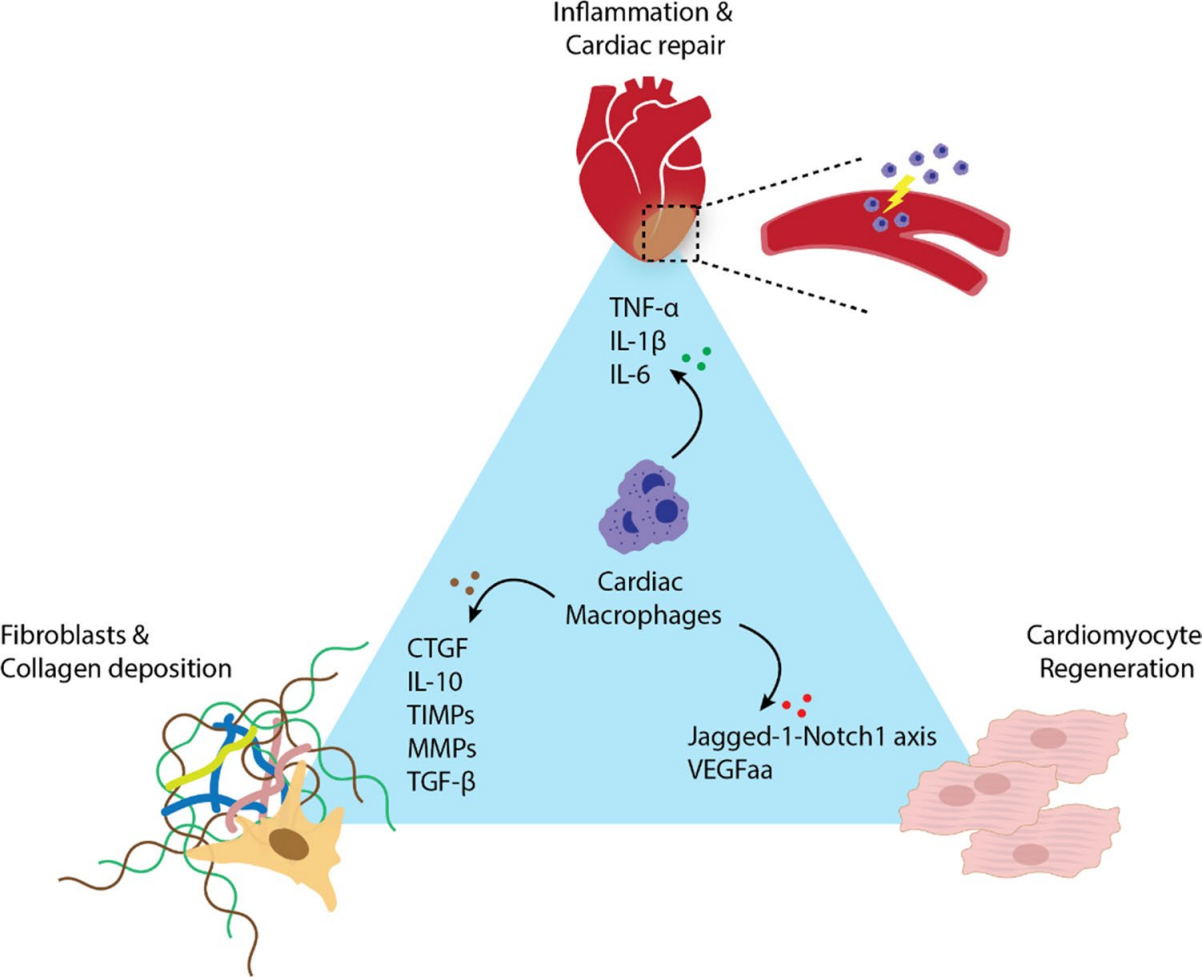
The role of cardiac macrophages in CVDs. Cardiac macrophages play pivotal roles in the progression of cardiovascular diseases (CVDs), encompassing inflammation, fibrosis, cardiac repair, and regeneration through the secretion of mediators and interactions with other cardiac cells. *TNF-α* tumor necrosis factor alpha, *IL* interleukin, *CTGF* connective tissue growth factor, *TIMP* tissue inhibitor of metalloproteinases, *MMP* matrix metalloproteinases, *TGF-β* transforming growth factor-β, *Notch 1* neurogenic locus notch homolog protein 1, *VEGFaa* vascular endothelial growth factor Aa

Due to the limited regenerative capacity of cardiomyocytes in adult heart, current therapeutic options are focusing in two directions: (i) increase regeneration of cardiomyocytes by stem cell therapy [11]; (ii) prevent adverse remodeling of non-myocytes including inflammation or fibrosis [12]. In this regard, macrophages have garnered significant attention in the last decades as attractive therapeutic targets for CVDs. Specifically, in the context of CVDs and HF, macrophages play active roles in all phases of the wound healing process following injury, ranging from inflammation to fibrosis and tissue remodeling [13]. Emerging studies have demonstrated the therapeutic potential of strategies aimed at modulating the activity of macrophages to prevent the development of pathological conditions associated with HF (Fig. 1).

### Inflammation

Macrophages are central players in the inflammatory response during progression of several types of CVDs [14, 15]. Upon cardiac damage, necrotic cells secret pathogen/damage-associated molecular patterns (PAMPs/DAMPs), which are the main triggers for the activation of immune cells including macrophages. The released PAMPs/DAMPs are then recognized by pattern recognition receptors (PRRs) on macrophages, eventually activate macrophages and initiate the inflammatory signaling cascades. Macrophages undergo polarization states during the progression of several CVDs and this status is highly associated with an inflammatory response. In the early stages of CVD, classically activated macrophages (simplified as M1 macrophages) are actively recruited into damaged myocardium [7]. They produce and release various pro-inflammatory cytokines, chemokines, and enzymes that contribute to tissue inflammation. Excessive and sustained inflammation can lead to tissue damage, impaired healing, and adverse cardiac remodeling. Over time, as the disease progresses and inflammation persists, macrophages can polarize to alternative-activated macrophages (also known as M2 macrophages). These M2 macrophages initiate a reparative healing process by releasing of anti-inflammatory cytokines. This process involves resolving inflammation and promoting tissue repair, which eventually results in decreased adverse remodeling and tissue damage [7]. However, in the context of prolonged and chronic inflammation in CVDs, the M2 polarization often becomes impaired, resulting in an imbalance between pro-inflammatory and anti-inflammatory response and contributing to HF progression [16]. Aging could also serve as a significant link between chronic inflammation and CVD risk. Notably, age-related DNA mutations that lead to a bone marrow abnormality, called clonal hematopoiesis of indeterminate potential (CHIP), can further exacerbate this imbalance by disrupting macrophage function. Specifically, mutations in TET2 and DNMT3A, two genes frequently mutated in CHIP, disrupt the delicate balance of cytokines that control macrophage behavior, leading to excessive inflammation. This further promotes the development of atherosclerotic lesions and increases CVD risk, highlighting the importance of macrophages in regulating inflammation [17].Therefore, modulation of macrophages holds promise as a therapeutic approach in CVDs. Strategies aimed at promoting the transition of macrophages from pro-inflammatory (M1) to anti-inflammatory (M2) can help mitigate chronic inflammation and hinder HF progression. Potential approaches will be discussed in further sections.

### Fibrosis

Followed by resolution of inflammation, the viable part of the damaged cardiac tissue initiates extracellular matrix (ECM) remodeling to compensate for the loss of cardiomyocytes. This stage, known as fibrosis, is distinguished by an excessive accumulation of collagen and other components of the ECM, primarily attributed to the heightened activation of fibroblasts. Such aberrant deposition results in increased stiffness and irregular cardiac rhythm, ultimately leading to sudden cardiac death in individuals with CVDs. As being a key player in inflammatory response, macrophages are also indispensable effector cells during development of the cardiac fibrosis through regulating multiple pathways related to activation of fibroblasts, such as TGF-β pathway or metalloproteases (MMPs) [18]. Significant insights arising from the single-cell sequencing investigation of cardiac cells encompass the identification of potential interactions between macrophages and fibroblasts mediated by ligand-receptor signaling circuits. Cell–cell interactions have been shown to predict a co-expression pattern of fibronectin 1 (FN1), lymphatic vessel endothelial hyaluronan receptor 1 (LYVE1), cluster of differentiation 74 (CD74), and macrophage migration inhibitory factor (MIF). This observation suggests that LYVE1 + monocytes and macrophages may interact with fibroblasts via the CD74-MIF pathway, implying a role for this interaction in the progression of fibrosis development [19, 20]. Disruptions in these interactions can result in either exaggerated fibrosis or defective fibrosis, both of which can have detrimental consequences for tissue repair and functional outcomes of the heart. The reciprocal regulatory influence of macrophages on fibroblasts and cardiac fibrosis is also highly associated with distinct subpopulations of macrophages [21]. Alternatively activated macrophages, also known as M2 phenotype, play a predominant role in wound healing and tissue remodeling. However, they also significantly contribute to cardiac fibrosis by directly promoting collagen I expression [22]. Consequently, inhibiting M2 macrophages through GATA3 depletion during fibrosis initiation has demonstrated cardioprotective effects and limited fibrotic injury in mouse MI model. However, manipulating macrophage populations demands careful consideration of their double-sword functional roles during the healing process. Depletion of M2 macrophages during resolution phase, the stage dedicated to inflammation resolution and tissue repair, results in worsened cardiac function along with exaggerated inflammation [23, 24]. This highlights that precise timing is crucial when implementing macrophage modulation strategies, ensuring alignment with their functional contributions at each stage of healing.

Additionally, specific subpopulations of M2 macrophages exhibit contrasting effects on fibrosis development in Coxsackievirus-induced myocarditis [25]. TLR4^+^casp-1^+^IL-1β^+^M2 macrophages promote fibrosis and inflammation, while Tim-3^+^M2 macrophages tend to exhibit an anti-fibrotic and anti-inflammatory phenotype [18, 25]. Furthermore, activated macrophages serve as the primary source of inflammatory cytokines such as TNF-α, IL-1, which can act as pro-fibrotic factors, promoting excessive ECM proteins production. Ultimately, this process results in poorly ordered ECM deposition and impaired tissue remodeling [26, 27]. It is important to note that macrophage phenotypes and their fibrotic effects can be influenced by various factors, including the specific pathological conditions, the stage of fibrosis, and the interplay with other cells and signaling molecules [28]. Therefore, investigating the functional properties of distinct macrophage subsets and elucidating the mechanisms governing macrophage phenotypic transitions, differentiation, and recruitment holds promise for developing strategies to attenuate and reverse pathological cardiac fibrosis.

### Tissue repair

Excessive fibrosis has a further impact on the chronic remodeling of the injured heart including scar and larger vessel formation, ultimately contributing to overall long-term survival and cardiac function. These changes consequently influence on the overall microenvironment cue and the fitness of other cardiac cells such as cardiomyocytes and endothelial cells. In this context, macrophages emerge as crucial contributors to the reparative cellular responses. More importantly, macrophages play a direct role in the cardiac conduction system through their interaction with cardiomyocytes. Macrophages residing in sinoatrial (SA) node and atrioventricular (AV) node establish electrical coupling with adjacent cardiomyocytes through gap junction, which allow for direct transfer of electrical signals between cells, enabling the syncronized propagation of electrical impulses and ensuring efficient conduction throughout the heart [29]. While their specific role in cardiac repair following injury remains to be fully elucidated, macrophages undoubtedly play a critical role in preventing abnormal cardiac rhythms, which can lead to sudden cardiac death in the injured heart. By aiding in removal of debris and necrotic cells, they involve in maintenance of mitochondrial health and cardiac homeostasis. Moreover, they secret cardiomyocytes protective molecules such as FGF-1, TNF-α, and IGF-1 to maintain survival of cardiomyocytes [30]. Indeed, depletion of macrophages has been shown to result in cardiac rhythm disturbance, suggesting their essential role in maintaining cardiac health under stress conditions [31]. However, considering diversity and phenotypic plasticity, macrophages can be also served as a cellular source for promoting pathological pathways through secretion of inflammatory cytokines. Macrophages secrete cytokines such as IL-6 or IL-8, which subsequently trigger hypertrophy of cardiomyocytes. Notably, the neutralization of these cytokines has been shown to mitigate hypertrophy, highlighting their significance in this regulatory process [32].

Macrophages also play a vital role in maintaining vascular health and structural integrity. In vitro studies have demonstrated that macrophages enhanced angiogenesis, the process of new blood vessel formation, by stimulating the proliferation and vessel sprouting of ECs. This angiotrophic potential is further amplified when macrophages are polarized towards an inflammatory state by treatment with lipopolysaccharide (LPS) [33]. Different macrophage phenotypes exhibit distinct functions in vascular remodeling. Inflammatory monocytes and macrophages (Ly6C^+^, CCR2^+^) are extravasated into peripheral inflamed tissues, contributing to the vessel inflammation and formation of atherosclerotic plaque [34, 35]. Conversely, the opposing population, including Ly6C^low^ or Cx3cr1, Nr4a1 macrophages, are enriched within capillaries and scavenge microparticles from their luminal side and function as intravascular housekeepers for fitness of endothelial cells by promoting the safe disposal of ECs and facilitating appropriate cell turnover [36, 37]. Overall, balancing between cardioprotective and detrimental subpopulation of macrophages at the appropriate healing stages for each pathological condition is pivotal to ensure enduring therapeutic effects while minimizing secondary tissue damage.

### Regeneration

The loss of cardiomyocytes following cardiac injury is irreversible. Due to limited regenerative capacity, the majority of current therapies are focused on either reducing damage on myocardium or recovering cardiomyocytes by stem cell therapy. However, these approaches face limitations due to low efficiency or high cost. Unlike in adult heart, neonatal heart has the stunning capability to reverse injury by regeneration of myocardium. Lavine et al. discovered distinct composition of macrophages between adult and neonatal cardiac tissues, both at baseline and following injury. The adult heart exhibited a coexistence of MHC-II^low^ and MHC-II^high^ subsets of macrophages (CCR2^−^) and monocytes (CCR2^+^), whereas neonatal hearts exclusively contained MHC-II^low^ macrophages and monocytes. These distinctive variances are associated with distinguishing outcome in cardiac remodeling post-injury [38]. In response to injury, neonatal hearts exhibit a selective expansion of resident macrophages population (MHC-II^low^CCR2^−^), which play critical roles in cardiac regeneration. These roles encompass the regulation of inflammation and the promotion of cardiomyocyte proliferation via Jagged-1-Notch1 axis, ultimately culminating in improved cardiac recovery post-injury [39]. Conversely, the adult heart experiences the replacement of these resident macrophages primarily by monocyte-derived macrophages that lack regenerative functions. Instead, they tend to foster a pro-inflammatory response, often leading to hypertrophy or fibrosis. Apparently, cardiac-resident macrophages (CRM) undergo a switch in their ontogeny after birth, from regenerative to adult-like phenotypes, resulting in the loss of proliferative potential [39]. Notably, preservation of resident macrophages through the inhibition of monocyte-derived macrophage recruitment has been demonstrated to yield minimal inflammation while fostering enhanced cardiomyocyte renewal in the adult heart [40]. Concordantly, studies in regenerative zebrafish also have provided compelling evidence for the indispensable role of macrophages in heart regeneration, notably by facilitating the self-renewal of cardiomyocytes. Particularly, macrophage-dependent expression of Vegfaa on epicardial cells has shown a robust association with cardiac growth signaling pathway and proliferation of cardiomyocytes [41]. The study by Rotem et al. revealed that the secretion of cytokines by macrophages plays a crucial role in determining the healing outcome of injured hearts in both neonates and adults. Among the various cytokines secreted, neonatal cardiac macrophages release high levels of osteopontin (OPN) following MI. Notably, OPN deficiency in cardiac macrophages impaired their paracrine reparative properties, highlighting the importance of macrophage-derived OPN in myocardial healing in neonatal hearts. Mechanistically, OPN secretion is essential for neonatal regeneration by stimulating cardiomyocyte cell-cycle re-entry and activating the proliferation and migration of non-CM cells. Remarkably, myocardial injection of recombinant OPN into adult hearts significantly improved infarct healing and overall cardiac function following MI, suggesting that the regenerative potential of neonatal macrophages can be harnessed to develop therapeutic strategies for acute MI in adult hearts [42].

These significant findings underscore the potential of reprogramming adult macrophages to acquire a regenerative phenotype, thereby opening up novel therapeutic avenues for treating cardiovascular diseases. The implications of these discoveries hold great promise for the field of regenerative medicine and present an exciting trajectory for future research in this domain.

## Patient selection and target identification for macrophage-based therapies in pathological conditions

The benefits of macrophage-targeted therapy in CVD patients, particularly those with heart failure, can vary depending on the specific therapeutic approach and the individual patient's condition. However, individuals with chronic heart failure, especially those with evidence of persistent inflammation, excessive fibrosis, and impaired tissue repair, are more likely to benefit from such therapies. It's important to note that macrophage modulation should be carefully tailored to the specific pathological context, as macrophages have diverse functions (due to their plasticity, macrophages can exhibit both protective and pathogenic functions) and their complete abrogation or excessive suppression can have unintended consequences.

### Atherosclerosis

Atherosclerosis is a chronic inflammatory condition characterized by the buildup of apolipoprotein B-containing lipoproteins and plaques within the arterial wall. It has garnered significant attention in the context of therapies targeting macrophages [43]. In the immune landscape of atherosclerotic plaques, macrophages, along with T-cells, represent the predominant cell types. Macrophages constitute approximately 50% of CD45^+^ cells within murine aorta upon atherosclerosis, but their prevalence decreases to 16–20% of total CD45 + cells in human carotid endarterectomies, indicating notable disparities of myeloid cell populations between humans and mice in atherosclerosis [44, 45]. Despite this difference, both pro-inflammatory and anti-inflammatory macrophage subtypes coexist within the plaque region in both species. In the pathogenesis of atherosclerosis, macrophages release a plethora of pro-inflammatory mediators, as well as anti-inflammatory factors, pro-thrombotic tissue factors, and matrix-degrading proteases. These molecular agents collectively exert significant influence over plaque growth, cellular composition, and stability. Several single-cell studies examining atherosclerotic plaques in both human and mouse models (e.g., Apoe-/- or Ldr-/- mice, under either a standard diet or a high-fat westernized diet) have unveiled the presence of three primary macrophage populations. These populations exhibit distinct gene expression signatures relevant to atherosclerotic disease, encompassing inflammatory macrophages, resident-like macrophages, and lipid-associated Trem2^hi^ macrophages [46, 47].

#### Inflammatory macrophages

In response to an inflammatory environment, monocytes are recruited and differentiate into macrophages within atherosclerotic lesions [43, 48]. Inflammatory macrophages are one of the major macrophage populations within atherosclerotic aorta. Inflammatory macrophages, also known as chemokine^high^ macrophages, are highly associated with atherosclerotic plaque progression. These macrophages secrete pro-inflammatory markers, digest lipoproteins, and accumulate foam cells, a hallmark of atherosclerotic lesions. This contributes to plaque growth and the release of inflammatory mediators such as IL-6 and TNF-α [43, 48]. The activated pro-inflammatory macrophages exhibit the expression of surface markers including major histocompatibility complex class II (MHCII), Fc receptor CD64, CD80, and CD86, along with the upregulation of typical pro-inflammatory transcripts such as Cxcl2, Ccl4, interleukin-1β (IL-1β), tumor necrosis factor alpha (TNF-α), NLR family pyrin domain containing 3 (NLRP3), caspase-1 (Casp1), and caspase-4 (Casp4) [49]. Inflammatory macrophages are typically enriched in non-foamy cells localized at the plaque shoulder region, which is normally mediated through inflammatory pathways, including Toll-like receptor (TLR) activation, TNF signaling, type I interferon (IFN) responses, and cytokine-chemokine interaction signaling pathways [50, 51].

#### Resident-like macrophages

Recent research findings reveal that the predominant myeloid cell population within the intima of the aortic arch consists of resident macrophages, which rely on CSF1 (colony-stimulating factor 1) expression and are sustained by local proliferation mechanisms. However, during the progression of atherosclerotic plaques, this resident population is gradually supplanted by recruited monocytes, primarily due to their limited capacity for self-renewal [52]. They are newly recognized population, called monocytes derived resident-like macrophages by Willemsen and Winther et al. [46]. In murine model of atherosclerosis, the resident-like macrophages primarily exhibit gene expression pattern similar to resident macrophages, including Cx3cr1, Clec4a2 (C-type lectin receptor), Lyve1, Mrc1, Forl2 and Vsig4, F13a1 [47, 53, 54]. Interestingly, atherosclerotic resident-like macrophages expressed high level of CCR2, a marker typically associated with recruited macrophages, supporting that recruited monocyte-derived macrophages replenish the resident-like macrophages upon atherosclerosis development [46, 50]. Moreover, this population express CD206, Folr2, Cbr2 and Selenoprotein-1 (Sepp1), all of which are associated with M2-like phenotype, determining the anti-inflammatory features of atherosclerotic resident-like macrophages [55]. Notable, the CD206^hi^CD163^hi^ macrophages in human atherosclerotic plaques resemble to the resident-like macrophage subsets described in murine atherosclerosis [44, 51]. In contrast to a detrimental role of the inflammatory macrophages, the resident-like macrophages exhibit protective functions against atherosclerosis progression by enhancing homeostasis. During atherogenesis in mouse model, depletion of these resident-like macrophages resulted in dysregulated cholesterol metabolism, which further led to exacerbated disease progression, characterized by increased arterial stiffness and collagen deposition, thus reinforcing the significance of the resident-like macrophages in maintaining fitness of heart against atherosclerosis [53, 54]. The resident-like macrophages are mainly involved in receptor-mediated endocytosis and proliferation signaling pathways [49, 50].

#### Foamy Trem2^hi^ macrophages

The third subpopulation, Trem2^hi^ macrophages, corresponds to lipid-laden foamy macrophages, which are recognized as principal trigger for atherosclerotic plaque formation. These macrophages are notably characterized by an elevated expression of key markers, such as Trem2, CD9, Fabp4, Apoe and Apoc1, and they are predominantly localized within the plaque intima and its necrotic core [46]. Fabp4 serves as reliable marker for identifying foamy macrophages within atherosclerotic lesions. CD9 is also known to play a pivotal role in foam cell formation through its association with CD36, a well-known stimulator of foam cell development [46]. In obese patients, CD9^hi^ adipose tissue macrophages demonstrate a higher intracellular lipid content in comparison to CD9^lo^ adipose tissue macrophages [56]. Particularly, Trem2^hi^ macrophages are only found in atherosclerotic plaque, not in healthy aorta as shown by the findings from Cochain et al. [49]. A pathway analysis of these Trem2^hi^ macrophages reveals their enrichment in processes related to lipid metabolism, regulation of cholesterol efflux, and oxidative stress, underscoring the involvement of Trem2^hi^ macrophages in intracellular lipid accumulation and foam cell generation [49]. More interestingly, these macrophages also present pro-fibrotic characteristics, as evidenced by the expression of Galectin-3, which may indicate a potential role of Trem2^hi^ macrophages in stabilizing plaques macrophage populations, drivers for plaque progression [57, 58]. Interestingly, these subpopulations display diminished pro-inflammatory phenotypes, hinting at an anti-inflammatory nature of Trem2^hi^ macrophages. Similarly, Trem2 is typically expressed on anti-inflammatory macrophages and has been demonstrated to restrain macrophage activation [59].

### Ischemic heart disease and myocardial infarction (MI)

Atherosclerosis can further provoke secondary complications. The progressive enlargement of atherosclerotic plaque can result in the occlusion of the vessel lumens, leading to ischemic injury or MI, one of the leading causes of death worldwide [60]. MI is a clinical condition characterized by the death of cardiomyocytes and tissue damage due to impaired oxygenation of the myocardium. The loss of cardiomyocytes concomitant changes in cell proportion, which lead to inflammatory response and cardiac remodeling in ischemic myocardium [13]. This pathological condition, commonly known as a heart attack, is another CVD that can potentially benefit from macrophage-based therapies.

Macrophages are the most abundant cell population among leukocytes infiltrated following MI, constituting 58.7% of the total CD45 + cells [61]. They are dynamically changed over the MI time continuum and play pivotal roles in balancing inflammatory response. The proportion of macrophages drastically dropped on day 1 (24.9%), but gradually recovered from day 3 (66.8%), peaking on day 7 (84.0%) post-MI [61]. More interestingly, macrophages exhibit temporal diversity in phenotypes during disease progression following MI. The polarization status of macrophages largely determines their pathological functions and impact during different phases of MI. In a murine model, within the early phase after MI (1–3 days post-MI), macrophages are actively recruited to the site of injury and initiate the clearance of necrotic tissue. During this phase, macrophages also release pro-inflammatory cytokines, which can intensify the inflammatory response. Once necrotic debris is removed, at later phase of MI (5–7 days up to months post-MI), anti-inflammatory macrophages replace the pro-inflammatory macrophages paving the way to the wound healing phase and resolution of inflammation. During this latter, macrophages release anti-inflammatory cytokines which stimulate the release of collagen via the TGF-β cascade, promoting scar formation [62, 63]. This transition of macrophages from a pro-inflammatory to a reparative phenotype should occur at the appropriate timing for the proper resolution of inflammation.

#### Early pro-inflammatory macrophages

Upon ischemia injury following coronary occlusion, remarkable infiltration of macrophages was observed from day 1 and persisting through day 7 post-operation [64]. Several previous scRNA-seq analyses have unveiled distinct subpopulations of cardiac macrophage during MI progression. In murine model of MI, cardiac macrophages were further subdivided into seven distinct subclusters, comprising three tissue-resident macrophages and four ischemia-associated macrophages. Among them, Olr1^+^ macrophages, one of ischemia associate macrophage populations, were the largest population at day 1 post-MI, accounting for 15% of total CD45 + cells. These Olr1^+^ macrophages exhibit pro-inflammatory characteristics and are endowed with phagocytic capabilities [64]. This finding aligns with the transcriptomic profiles of isolated cardiac macrophages post-MI, affirming that the early macrophage response following MI is marked by pro-inflammatory phenotypes [65]. In a separate study by Jin et al., the most signature genes characterizing inflammatory macrophages in the context of MI are identified as CCR2 and MHC II. CCR2^hi^ macrophages represent infiltrating monocyte-derived macrophages, predominantly expressing canonical macrophage markers like Cd68, Fcgr1, Itgam and CCR2. This subset also peaked in abundance on day 1 and gradually decreased until day 7 post-MI [61, 64]. MHC^high^ subset is notably enriched with antigen processing and presentation-related genes, primarily exhibiting pro-inflammatory functions [66]. Associated signaling pathways of these post-MI inflammatory macrophages encompass NF-kB and NOD-like receptor signaling pathways, which are closely linked to activated leukocyte migration and cytokine productions, featuring by upregulated expression of Cxcl2, Ccl9, Ccl24, Il1b, and Trem1 [64].

#### Late reparative macrophages

Subsequent to MI, macrophages at later stages exhibit a relatively higher gene expression of Apoe, Fcrls, Rgs10, Adgre1, Trem2, Gpnmb, Fabp5, Spp1 and Timp2. Their expression gradually increases, becoming most prominent at 7 days post-MI, with potential extension over subsequent months [61]. In particular, Gpnmb^+^ macrophages exhibit a notable surge in infiltration at 7 days post-MI, accompanied by elevated phagocytic activity. They display enrichment of lysosome and cholesterol metabolism pathways, marked by increased expression levels of Gpnmb, Fabp5, and Trem2 [64]. Interestingly, the late macrophages demonstrate a distinctive metabolic profile compared to early macrophages post-MI. Late Gpnmb^+^ macrophages exhibit increased phagocytic and fatty acid oxidation scores, whereas early Olr1^+^ macrophages show elevated senescence‐associated secretory phenotype (SASP) and glycolysis scores [64]. As similarly observed in atherosclerosis, Trem^hi^ macrophages are present in advanced infarct region following MI, with predominant expression in the later stage [61]. This subpopulation is marked by anti-inflammatory characteristics, evidenced by high expression of anti-inflammatory genes such as Arg1, IL-10 and Tgfb1. Of particular significance, Trem2^hi^ macrophages also display a heightened expression of osteopontin (Spp1), which is related to pro-fibrotic potential in regulating post-MI LV remodeling [61]. In line with these findings, a recent study from Kim et al. reported the therapeutic potential of a molecule secreted by Trem^hi^ macrophages, soluble Trem2 (sTrem2). In vivo, administration of sTrem2 significantly improved myocardial function and LV remodeling post-MI by promoting polarization of macrophage toward an anti-inflammatory phenotype, which results in effective regulation of inflammation in the infarcted myocardium [67].

#### Resident macrophages (steady-state macrophages)

In contrast to infiltrated macrophages, tissue-resident macrophages have been comparably less studied and were often considered of insignificance during MI progression. However, in recent years, there has been a growing awareness of their role [68, 69].

Zhuang et al. recently identified three tissue-resident macrophage subsets in the heart following MI using scRNA-seq [64]. Among those, the Timd4 + cluster represents the most conserved tissue-resident macrophage subset across multiple organs in both mice and humans [70]. This subset is enriched in lysosome and endocytosis signaling pathways and exclusively expresses Folr2, Timd4, and Lyve1 [61, 71]. Tissue-resident macrophages are primarily sustained through local proliferation, as evidenced by elevated expression of proliferation-related genes such as Jund, Tcf4, and Maf [71]. Although their relative proportion declines on the first-day post-MI, it gradually restores by day 7, yet does not return to steady-state levels. This suggests an indirect implication of their involvement during the later stages of MI. A central role of these macrophages is to attenuate the post-MI inflammatory response [71, 72]. Depletion of resident macrophages deteriorates cardiac function and impairs healing after MI, highlighting their protective role. Resident macrophages are essential for efficiently clearing necrotic and apoptotic debris from the infarcted area, which contributes to timely inflammation resolution. Additionally, pro-inflammatory signaling pathways, such as NF-κB, apoptosis, and IL-17, are significantly less pronounced in Folr2 + resident macrophages than in Folr2- monocyte-derived macrophages [71]

### HFpEF (heart failure with preserved ejection fraction) and diastolic dysfunction

Unlike systolic dysfunction, HFpEF is characterized by impaired LV contractility and diastolic relaxation, while the overall function of cardiomyocytes remains preserved [73, 74]. Unfortunately, conventional pharmacological interventions employed against heart failure with reduced ejection fraction (HFrEF), such as beta blockers or ACE inhibitors, have proven ineffective in restoring cardiac function in HFpEF patients. Despite its growing prevalence, the specific treatment for HFpEF is still limited. Emerging studies reveal the significant role of inflammation in cardiac remodeling for both HFrEF and HFpEF, emphasizing the need for distinct therapeutic approaches due to distinctive pathophysiology [75]. Notably, HFpEF exhibits more pronounced systemic inflammation and fibrotic pathways. Myocardial biopsies from patients with HFpEF reveal a higher number of infiltrated inflammatory cells than those from healthy controls. These inflammatory cells play a crucial role in the intense inflammatory response and secretion of pro-fibrotic growth factors such as TGF-β [76]. This pattern is also evident in the blood of HFpEF patients, where there is a two- to fourfold increase in classical, intermediate, and non-classical monocyte subsets, indicative of a chronic state of inflammation during HFpEF [77]. Consequently, significantly elevated systemic levels of inflammatory cytokines, such as TNF-α, IL-6, and the chemokine CCL2, have been observed in HFpEF patients experiencing disease exacerbation, with increases ranging from 1.3- to 2.4-fold compared to those with stable disease [78, 79]. Interestingly, these hematopoietic activities are closely correlated with myocardial filling pressure [77]. This underscores the role of systemic inflammation as a potential trigger for the development of fibrosis by promoting the activation of myofibroblasts. The amplified fibrotic response ultimately leads to diastolic dysfunction, thereby contributing to clinical deterioration in patients with HFpEF [77].

#### Profibrotic macrophages

Macrophages are among the major effector cells in the inflammatory response. While the contribution of macrophages on HFpEF progression is comparably less understood than in HFrEF, emerging evidence has linked macrophages, particularly pro-fibrotic M2 macrophages, to adverse outcomes in HFpEF [77, 80]. In contrast to their beneficial effects in HFrEF by resolving inflammation and promoting wound healing, these M2 macrophages exhibit a more pathological role in HFpEF, manifesting an enhanced fibrogenic phenotype. In HFpEF, M2 macrophages play a critical role in myofibroblast activation and collagen deposition by secreting fibrosis stimulators like Galectin-3 or TGF-β, ultimately leading to myocardial stiffness and diastolic dysfunction [77, 80, 81]. Significantly, the IL-10 signaling pathway plays a central role in driving the polarization of macrophages towards a pro-fibrotic phenotype during the progression of HFpEF. Macrophage-derived IL-10 secretion creates an autocrine loop that promotes pro-fibrotic macrophage polarization and the release of fibroblast-activating molecules such as TGF-β and osteopontin [77]. Remarkably, HFpEF patient-derived serum enhances the differentiation of healthy monocytes into macrophages that express IL-10, indicating that prolonged exposure to the HFpEF patient's microenvironment directs macrophages towards a fibrogenic phenotype. These findings highlight the potential of IL-10 as a therapeutic target for regulating pro-fibrotic macrophages and mitigating HFpEF complications, including myocardial collagen deposition and diastolic dysfunction [77, 80, 81]. This contrasts with the pattern observed in HFrEF, where IL-10 is beneficial for tissue repair and inflammation resolution [82]. These findings suggest that the same macrophage pathway can have either beneficial or detrimental effects depending on the stage of disease progression and the concomitant changes in the myocardial microenvironment. Such insights have important implications for developing targeted therapeutic strategies to limit disease progression in HF of various etiologies. However, to achieve optimal and efficient therapeutic outcomes, a comprehensive understanding of macrophage behavior and function during the progression of HFpEF is needed.

## Current status of macrophage-targeting strategies

In the last years, several attempts have been applied to fine-tune the biology of macrophages. In this section, we illustrate the most promising and challenging therapeutic approaches aimed to modulate the chemotaxis, inflammatory response as well as phenotype of these phagocytic cells (Fig. 1).

### Atherosclerosis

#### Depletion of monocytes/macrophages

The negative prognostic significance of macrophage infiltration and their persistence in atherosclerosis supports the development of treatment options, which includes either blocking the recruitment of macrophages or neutralizing relative pro-inflammatory cytokines. Depletion of macrophages has emerged as an effective strategy, particularly for individuals with atherosclerosis, owing to the strong association between recruited monocytes/macrophages and the growth and destabilization of atherosclerotic plaques, which typically exhibit a large lipid core and a weakened fibrous cap [83, 84]. Pharmacological depletion of macrophages can be achieved by promoting programmed cell death, either through enhanced apoptosis or autophagy [85]. One extensively utilized method for in vivo investigation of macrophage function involves the use of clodronate liposomes (Clo-Lip). Clo-Lip depletes macrophages by instigating programmed cell death [86, 87]. Depletion of macrophages induced by Clo-Lip administration has been shown to significantly improve systolic blood velocity in atherosclerosis, underscoring the therapeutic potential of inhibiting macrophage accumulation to impede the progression of atherosclerosis [88]. However, chronic administration of Clo-Lip does present limitations. Delayed macrophage recruitment resulting from Clo-Lip intervention can lead to impaired neutrophil resolution and subsequent heart regeneration [89]. Additionally, a recent study by Culemann et al. has challenged prior notions by revealing that neutrophils are the primary effectors impacted by Clo-Lip, rather than monocytes or macrophages [90]. Clo-Lip exerts anti-inflammatory effects independently of macrophage presence, but its effectiveness is contingent on proper neutrophil function. However, the precise mechanisms through which Clo-Lip-induced impairment of neutrophil function influences monocytes or macrophages remain to be fully elucidated. This discovery prompts a re-evaluation of the intricate relationship between neutrophils and macrophages, with the goal of optimizing the use of Clo-Lip for inflammation regulation [90].

Extensive research has focused on disrupting specific chemokine-chemokine receptor pairs, such as CCL5–CCR5 and CCL2–CCR2, to impede monocyte and macrophage recruitment. Interventions involving CCL5 (RANTES) antagonists, CCL2 (MCP-1) inhibitors, or the silencing of CCR2 mRNA have shown significant reductions in macrophage infiltration within atherosclerotic plaques. These interventions have led to a reduction in lesion size and plaque stabilization [91–93]. However, the translation of these findings into clinical trials for agents targeting these chemokine axes has been lagging behind, primarily due to systemic inhibition and the complexity of these pathways, which can result in side effects on non-targeted tissues [94, 95]. Strategies to address this challenge include the optimization of drug delivery through tissue-specific nanoparticles or exosomes loaded with monoclonal antibodies, as well as the development of CCR2-biased or probe-dependent antagonists, which can selectively block specific chemokines or signaling pathways. These strategies hold promise for clinical implementation [94, 96]. Inhibition of macrophage migratory inhibitory factor (MIF), a cytokine-inducing adhesion molecule like ICAM1, and VCAM1, can also contribute to protective effects in limiting macrophage adhesion. Treatment with antibodies against MIF has resulted in a significant reduction in the number of infiltrated macrophages and foam cells, leading to improved cardiac function and reduced scar size [97, 98]. Statins, a class of cholesterol biosynthesis inhibitors, are widely prescribed for the treatment of atherosclerosis. Administration of statins effectively manages hyperlipidemia and results in a significant reduction in macrophage infiltration within atherosclerotic plaques within one month, supporting the clinical application of macrophage infiltration inhibition as a therapeutic strategy [99–101]. Similarly, interventions targeting the IL-1 pathway, such as Anakinra (IL-1R agonist; ClinicalTrials.gov, number NCT01950299, completed) [102, 103] or canakinumab (monoclonal antibody block IL-1β; ClinicalTrials.gov, number NCT01327846, completed) [104], have successfully transitioned into clinical studies. Their administration has led to a significant reduction in leukocyte production, resulting in limited adverse remodeling and attenuated systemic inflammation. This, in turn, has led to reduced mortality and hospitalization rates among patients with HF [102, 105, 106].

#### Induction of macrophage autophagy

As the role of macrophage-mediated inflammation at certain levels is essential for the wound healing process in various disease contexts, there is a growing interest in the modulation of macrophage behavior as an alternative to inhibition. This approach represents the next generation of macrophage-based therapeutic strategies. In the context of atherosclerosis, this approach is designed to promote plaque regression, reduce inflammation, and enhance overall plaque stability. One well-established strategy involves the induction of autophagy in macrophages [107]. Autophagy is a crucial cellular process responsible for removing and recycling damaged or long-lived intracellular materials. An increasing body of research has demonstrated the close link between autophagy and macrophage biology within the realm of innate immunity. Autophagy in macrophages plays a protective role in atherosclerosis, primarily due to its regulatory function in facilitating cholesterol efflux from macrophage foam cells [107, 108]. In the context of atherosclerotic plaque, the induction of autophagy in macrophages is largely dependent on the ATP-binding cassette transporter A1 (ABCA1)-mediated process [108]. Elevating ABCA1 levels in macrophages present a therapeutic effect by inhibiting the inflammatory response and progression of atherosclerotic lesions [109]. Selective inhibition of autophagy regulators, such as the PI3K/Akt/mTOR pathway, can effectively induce autophagy and reduce macrophage aggregation in atherosclerotic plaques, thereby promoting plaque stability by protecting cells and reducing the secretion of inflammatory factors [110]. Furthermore, autophagy serves as a regulator of efferocytosis, a process in which macrophages efficiently clear debris or necrotic cells, essential for organ repair following injury [111, 112]. Disruption of autophagy can lead to a loss of this function, resulting in prolonged inflammation, which in turn exacerbates atherosclerosis progression [113, 114]. Additionally, pro-reparative factors are secreted by macrophages after efferocytosis, further promoting wound healing [115].

#### Adipose tissue macrophages (ATM)

Being a lipid-driven chronic inflammatory disease, atherosclerosis is also closely linked to metabolic disorders such as diabetes or obesity [116]. Abnormal accumulation of pro-inflammatory adipose tissue macrophages (ATM) is a common feature in metabolic disorders, which can trigger the onset of other chronic diseases such as hypertension or cerebrovascular diseases [117]. The uncontrolled release of pro-inflammatory mediators like IL-6, IL-1β and TNF-α by ATMs plays a significant role in obesity-related adipose tissue inflammation and metabolic dysfunction [116, 118]. Therefore, the therapeutic options aimed at modulating the metabolic switch or shifting pro-inflammatory ATMs towards an anti-inflammatory phenotype hold promise in preventing atherosclerosis progression. These approaches attenuate inflammation, promote cholesterol clearance and improve plaque stability.

### Myocardial infarction

#### Macrophage polarization/inflammation resolution

Incomplete resolution of inflammation, characterized by prolonged and unresolved inflammatory processes, has been linked to the pathogenesis of adverse complications, potentially contributing to detrimental cardiac remodeling that ultimately leads to impaired cardiac function and heart failure development following MI [4, 114]. Macrophages, as crucial regulators of inflammation, have emerged as promising therapeutic targets for achieving a balanced pro- and anti-inflammatory response following MI [119–121].

Current therapeutic strategies that target macrophage activity in the context of MI primarily aim to promote the resolution of inflammation by increasing the presence of anti-inflammatory macrophages, while concurrently reducing pro-inflammatory signals and mitigating adverse remodeling [122]. This is possible by pharmacological interventions targeting related cell-signaling molecules, such as chemokines, cytokines, or antibodies. Well-established stimulants of M2 macrophages, such as IL-4 and IL-10, drive macrophages toward reparative phenotypes. This promotes post-MI tissue repair, which leads to ECM stabilization as well as improvement of cardiac function compared to control mice [103, 123, 124]. In this regard, transplantation of M2b macrophages has been demonstrated to significantly reduce fibrosis and prevent adverse remodeling in a mouse model of ischemia/reperfusion injury, providing strong evidence for the therapeutic potential of these cells in the context of cardiac remodeling [125]. Beyond the classical stimulators IL-4, IL-13, and IL-10, various factors capable of modulating macrophage polarization have been identified. These factors include Lrg4, Activator protein-1 (AP-1), cAMP-responsive element-binding protein (CREB) activation, and ECM components [126, 127]. AP-1 is a proinflammatory transcription factor complex composed of FOS and Jun family, which plays a role in regulating the inflammatory status by enhancing macrophage polarization [128, 129]. Consequently, inhibition of the AP-1/Fos signaling pathway has been demonstrated to have a protective effect on cardiac function following MI by promoting polarization of macrophage towards a more anti-inflammatory phenotype, resulting in enhanced resolution of inflammation [128, 129]. Similarly, Lrg4 can also govern pro-inflammatory macrophage activation by exerting synergy effects on AP-1 activation and subsequent CREB-mediated Fos transactivation. Macrophages lacking Lrg4 exhibit diminished inflammatory gene signatures. In alignment with these findings, macrophage-specific Lrg4 knockout mice demonstrated reduced ischemic injury as well as improved healing process, attributed in part to the regulation of AP activity [126]. In addition to cardiac macrophages, splenic monocytes/macrophages also play a detrimental role in myocardial ischemia/reperfusion (I/R) injury. Specifically, the NLRP3 inflammasome in splenic monocytes mediates an inflammatory response shortly after reperfusion, worsening MI/R injury in a mitochondrial cell-free DNA (mt-cfDNA)/TLR9-dependent manner. Notably, depletion of NLRP3 in splenic macrophages using CY09 administration effectively reduces infarct size [130]. Macrophages are major cell source of ECM components such as fibronectin or hyaluronic acid (HA) during tissue repair and wound healing. Interestingly, these by-products of ECM can also influence on phenotype switch of macrophages. In their recent study, Wang and colleagues reported that hyaluronic acid-derived short oligosaccharides (HA-o) decreased the infarct size and apoptosis as well as improved angiogenesis in post-MI mouse model, which is attributed to augmented M2 macrophages [131]. In another study, injection of recombinant collagen type I and type III matrices increased M2 macrophages by 1.5-fold at 28 days post-MI, limiting adverse remodeling as well as promoting healing environment [132]. While less well-known, epigenetic modification can also regulate macrophage polarization. In mouse models of atherosclerosis and MI, HDAC (Histone Deacetylase 9) inhibition promoted reparative macrophage polarization and reduced inflammation, suggesting a role for HDACs in macrophage activation [133, 134].

The therapeutic targeting of M2 macrophages in the context of MI is an intriguing approach, but it comes with certain limitations. An overabundance of M2 macrophages might contribute to excessive fibrosis in the post-MI heart through the activation of myofibroblasts, ultimately leading to impaired cardiac function. Particularly, M2 macrophages encompass four subsets: M2a, M2b, M2c and M2d, which are often referred to collectively as M2 macrophages. While all M2 macrophages subtypes possess immunosuppressive properties, they exhibit distinct expression markers and functions. For example, M2a and M2c subtypes presented more pro-fibrotic phenotype, whereas M2b is considered regulator cells [125]. Moreover, M2 macrophages are effective in reducing pro-inflammatory signals, which may potentially hinder efficient wound healing, since a certain degree of inflammation is necessary to facilitate the clearance of necrotic or apoptotic cells in the infarct region. Therefore, ensuring the appropriate polarization of the right M2 subtype at the correct time and place is essential.

#### Resident macrophage survival

During the steady state of the heart, cardiac macrophages can be categorized into two major populations: embryonic monocyte-derived macrophages (CCR2^+^) and yolk sac-derived Cx3Cr1^+^CCR2^−^ resident macrophages. These distinctions are based on their origins and physiological characteristics, as determined through various fate-mapping techniques. In the human heart, there are also distinct macrophage populations that functionally parallel the roles of mouse cardiac CCR2^−^ and CCR2^+^ macrophages [135]. However, following cardiac injury, the balance between these two subtypes shifts significantly, with infiltrating circulating macrophages experiencing substantial alterations. Consequently, over the past few decades, the majority of research efforts have concentrated on understanding the dynamics of monocyte-derived macrophages. Only recently has the significance of self-renewing tissue-resident macrophages in pathological conditions gained greater recognition. A distinguishing feature of tissue-resident macrophages is their proficiency in phagocytosis, a process critical for the prompt removal of cellular debris resulting from injury. Additionally, they play a pivotal role in the maintenance of myocardial homeostasis by regulating tissue metabolic states [72]. The absence of resident macrophages, particularly those expressing Trem2, leads to impaired elimination of damaged mitochondria, resulting in heightened inflammation and myocardial dysfunction, particularly in conditions like septic heart disease [135, 136]. Similarly, their protective effects have been also presented in a murine model of MI, despite the significant loss of Cx_3_Cr1^+^ cells during maturation, with limited self-renewal capacity and regenerative potential, selective depletion of this population in adult hearts leads to impaired cardiac function and remodeling following ischemic cardiac injury, indicating that Cx_3_Cr1^+^ cells still play a role in wound healing in the adult heart [38, 71, 137]. Furthermore, cardiac-resident macrophages demonstrate robust proangiogenic and mitogenic properties, suggesting their potential in cardiac repair [138]. More interestingly, the injection of soluble triggering receptor expressed on myeloid cells 2 (Trme2), a crucial gene for the self-renewal capacity of CRM, results in improved cardiac remodeling and myocardial function following MI via polarization of macrophages toward anti-inflammatory phenotypes. This improvement is attributed to the polarization of macrophages toward anti-inflammatory phenotypes [61, 67]. Similarly, overexpression of CRM specifically expressing legumain (Lgmn) also improves cardiac function in mice after MI via efficient efferocytosis-mediated clearance of apoptotic cells [72]. These findings convey two key messages: 1. resident macrophages exhibit anti-inflammatory characteristics; 2. molecules associated with resident macrophage survival, such as Trem2, could serve as alternative therapeutic targets. Additionally, the specific role of CRM has gained attention in recent 4–5 years. The work by Hulsmans and colleagues highlights their role in facilitating electrical conduction of cardiomyocytes by interacting with connexin-43 gap junctions during steady-state conditions. While their role in disease contexts has been comparatively less explored, intriguingly, macrophage ablation has been linked to disrupted cardiac rhythm both in steady-state and after MI [139]. Moreover, the loss of resident macrophages leads to impaired ventricular remodeling and coronary angiogenesis, resulting in increased mortality in chronically failing hearts with reduced contractility [140]. These findings strongly emphasize the essential and protective role of resident macrophages in proper cardiac remodeling and maintaining cardiac function after injury. Importantly, these results underscore that not only inflammation-mediated infiltrating macrophages but also a reduced population of CRM can contribute to adverse remodeling and impaired cardiac function after MI. However, the underlying molecular basis for the distinct roles and responses to ischemic injury between circulating and tissue resident macrophages remains unclear. Future studies should aim to identify molecules or related signaling pathways to enhance the survival of CRM and their cardiac protective role while reducing inflammatory signaling in infiltrating monocyte-derived macrophages, ultimately optimizing cardiac remodeling.

### HFpEF (heart failure with preserved ejection fraction) and diastolic dysfunction

#### Inhibition of pro-fibrotic macrophages

HFpEF often manifests with LV diastolic dysfunction, primarily stemming from systemic inflammation and resultant interstitial fibrosis [73]. Although the role of macrophages in HFpEF progression is less extensively studied compared to in HFrEF, current research efforts have revealed that the crosstalk between macrophages and fibroblasts is inevitable in HFpEF pathogenesis [75]. Myocardial biopsies from HFpEF patients have shown a twofold increase in cardiac macrophage abundance and a 59% elevation in the gene expression of pro-fibrotic TGF-β compared to control samples. This heightened TGF-β appears to contribute to fibroblast activation and excessive collagen deposition [76, 77]. Consequently, inhibiting pro-fibrotic macrophages or their associated genes has demonstrated therapeutic potential in HFpEF and diastolic dysfunction [77, 141].

C–X–C chemokine receptor 4 (Cxcr4), a critical regulator of macrophage-mediated immune responses, is prominently expressed on infiltrated macrophages in both murine HFpEF models and patients with HFpEF. Cxcr4 + macrophages not only influence the inflammatory response but also play a role in myofibroblast transition via the activation of the Cxcl3–Cxcr2 pathway [142]. These effects can be reversed by myeloid-specific Cxcr4 deficiency, suggesting that inhibiting Cxcr4 may offer a novel therapeutic option to block macrophage Cxcr4 signaling and prevent cardiac diastolic dysfunction in HFpEF patients [142]. A study conducted by Hulsmans et al. identified IL-10 as the most up-regulated fibrosis-related genes in cardiac macrophages exposed to conditions such as salty drinking water, unilateral nephrectomy, and chronic exposure to aldosterone (SAUNA) induced diastolic dysfunction. Whereas inhibition of IL-10 in cardiac macrophages results in diminished fibroblasts activation and diastolic dysfunction [77]. The correlation between macrophage abundance/function and disease progression in human HFpEF underscores the development of therapeutics that inhibit macrophage recruitment and neutralize their detrimental inflammatory functions to promote recovery of the failing heart [75, 77]. In clinical trials, administration of Anakinra, the recombinant form of the naturally occurring IL-1 receptor antagonist, successfully attenuated both systemic inflammation and disease symptoms [143]. However, the specific macrophage subtypes involved in HFpEF and their precise roles, as well as the associated signaling pathways, remain areas of ongoing research and require further understanding for effective targeting.

### Non-coding RNA (ncRNA)

In recent decades, there has been a growing interest in developing innovative therapeutic strategies based on new scientific findings (Table 1). The rapid development of RNA-based approaches in basic research has inspired their application to clinical research. Within this context, the expanding knowledge surrounding various classes of non-coding RNAs (ncRNAs) and their diverse functional roles has sparked considerable enthusiasm as promising candidates for RNA interference (RNAi)-based gene regulation. The understanding of novel regulatory mechanisms involving ncRNAs and their substantial implications in the pathophysiology of numerous disorders and diseases has positioned ncRNA-based therapies as a focal point of interest. These therapies have shown great potential, particularly for addressing targets traditionally considered 'undruggable,' and for their prospective role in precision or personalized medicine across a spectrum of conditions, including CVDs [144]. By advance in transcriptomics analysis, several altered genes and ncRNAs have been determined during activation and polarization of macrophages [145].

**Table 1:** HF-associated macrophage-derived biomarker

Class	Subclass	Biomarker	Disease
Inflammatory mediators	Cytokines/Chemokines	TNF-α IL-6 IL-12 MCP-1 IL-1β	HFpEF [79, 80, 169]
	Peroxidase enzyme	MPO	CAD [68, 171]
Pro-fibrotic mediators	Growth factor	TGF-β GDF-15	HFpEF [175] AHF [177]
	Glycoprotein	Galectin-3	HFpEF [187, 188] MI risk stratification [185, 186]
Cell–cell network	Micro RNAs	miR-146a miR-155	Atherosclerosis [196] HF following MI [199]
	Gene expression	CUX1 CTSD ADD3	HF following MI [200]

*CAD* coronary artery disease, *AHF* acute heart failure, *HF* heart failure, *HFpEF* heart failure with preserved ejection fraction, *MI* myocardial infarction, *LV* left ventricular

#### miRNAs

MicroRNAs (miRNAs) are small (approximately 20–22 nucleotides) and among the most extensively studied ncRNAs. Recent studies have conducted a comprehensive miRNome analysis of major cardiac cell fractions, identifying several macrophage-specific enriched microRNAs (miRNAs). Among them, miR-21 is the highest expressed miRNA in cardiac macrophages both in health and disease (25% and 43% respectively of all miRNAs). MiR-21 is a key regulator of the profibrotic function of cardiac macrophages, which contributes to disease-associated fibrosis [146]. In a pressure overload-induced HF model of mice, macrophage-specific genetic deletion of miR-21 resulted in regulatory effects on fibrosis and cardiac dysfunction [146]. However, in contrast, miR-21 mimic is rather beneficial in an obstructed HF model. In vivo nanoparticle delivery of miR-21 to cardiac macrophages in the infarct area prompts the switch of macrophages towards a reparative phenotype, supporting tissue healing and curbing fibrosis [147]. These opposite effects of miRNAs describe that each pathological condition requires a proper strategy despite the common molecule. In an I/R mouse model, mesenchymal stromal cells (MSCs) generating miR-182 enriched exosomes are able to target macrophages and shift their polarization from pro- to anti-inflammatory phenotype, which results in decreased inflammation and infarct size [148]. More importantly, systemic depletion of macrophages using Clo-Lip abolished the therapeutic effects of MSC-Exo, suggesting the significant role of macrophages in mediating the effects of MSC-Exo [148]. MiR-33 is known to target genes involved in cholesterol homeostasis, including ATP binding cassette subfamily A member 1 (ABCA1) and PGC-1α in both human and mouse macrophages. This action limits cholesterol efflux from macrophages and promotes foam cell formation [149, 150]. Specific loss of miR-33 in macrophages reduces lipid accumulation and inflammation under hyperlipidemic conditions, resulting in reduced plaque burden [151]. MiRNAs such as miR-146a and miR-181b regulate macrophage polarization by their anti-inflammatory properties, thereby reducing the inflammatory response and atherosclerotic plaque development. Consequently, the targeted delivery of these miRNAs to macrophages mitigates inflammation and stabilizes atherosclerotic plaques [152, 153]. Interestingly, miRNAs are not only involved in regulating macrophage phenotypes but the enzyme responsible for generating miRNAs, Dicer, is also implicated in macrophage activation [154]. A study by Wei and colleagues demonstrated that specific ablation of Dicer in macrophages accelerated atherosclerosis in mice. This was accompanied by an enhanced inflammatory response and increased lipid accumulation in lesional macrophages. The miRNAs, generating Dicer, include miR-10a, let-7b, and miR-195a. Among them, miR-10a promotes fatty acid oxidation (FAO) in macrophages, which promotes the resolution of inflammation, limiting foam cell formation, and reducing atherosclerosis [154]. Notably, miR-10a expression was found to be negatively correlated with atherosclerosis progression in humans. Therefore, promoting Dicer/miR-10a signaling may represent a novel and promising therapeutic strategy for atherosclerosis. In human failing heart, miR-223-3p and miR-486-3p were identified as key drivers in the polarization of macrophages towards a pro-inflammatory phenotype [155], suggesting they can be potential therapeutic targets for HF via modulation of inflammatory states of macrophages.

#### Long non-coding RNAs (LncRNAs) and circular RNAs (CircRNAs)

Nevertheless, the modulation of macrophages can be mediated also by long non-coding RNA, which are longer than 200 nt, encompassing linear lncRNAs and circular RNAs (circRNAs).

RNA-seq profiling of atherosclerotic lesion intima has revealed the presence of a macrophage-specific lncRNA, called MAARS (Macrophage-Associated Atherosclerosis lncRNA Sequence) [156]. Targeted silencing of MAARS has been shown to significantly reduce atherosclerosis progression in a murine model by regulating macrophage apoptosis [156]. The expression of lncRNA SNHG16 is notably increased in both atherosclerosis patients and in ox-LDL-mediated atherosclerotic macrophages in vitro. Importantly, its elevated expression is associated with inflammation and foam cell formation via the NF-κB signaling pathway. Conversely, the inhibition of SNHG16 leads to opposing results, suggesting SNHG16 as a potential target for atherosclerosis treatment [157]. Another critical lncRNA involved in cholesterol metabolism in macrophages is MeXis, which plays a role in regulating ABCA1 expression. The loss of MeXis impairs macrophage Abca1 expression and accelerates atherosclerosis [158]. Like miRNAs, lncRNAs also impact macrophage polarization. For instance, NEAT1, whose levels were correlated with post-MI status, independent of statin intake and LV ejection fraction, is increased in ox-LDL-mediated atherosclerotic human THP-1 macrophages and promotes inflammation and lipid uptake [159, 160]. Whereas knockdown of NEAT1 in macrophages represses inflammation and the formation of foam cells, suggesting a role for NEAT1 in atherosclerosis development [160]. Another example is the lncRNA plasmacytoma variant translocation 1 (PVT1), which exhibits elevated expression in myocardial tissue and heart-infiltrating macrophages of sepsis mice. A major function of PVT1 is to enhance M1 macrophage polarization and promote LPS-induced myocardial injury via the miR-29a/HMGB1 axis. In murine model of lipopolysaccharide (LPS)-induced myocarditis, the knockdown of PVT1 prevents the polarization of macrophages toward a pro-inflammatory phenotype, which leads to relieved sepsis-induced myocardial injury [161].

CircRNAs represent a relatively less explored class of ncRNAs, and their role as miRNA- sponges is one of the well-established mechanisms of circRNAs in disease progression [162]. The interaction between circRNAs and miRNAs has also been observed in macrophages during the progression of CVD. For example, CircDENND1B has been identified to bind to miR-17-5p, exerting a pro-cholesterol-efflux function [162]. Increased level of circDENND1B in macrophages promotes Abca1-mediated cholesterol efflux, consequently reducing foam cell formation. This suggests that modulation of CircDENND1B/miR-17-5p axis in macrophages could be a potential therapeutic option for the regulation of foam cell formation in atherosclerosis [162]. Another circRNA, hsa_circ_0008896, was found to be significantly upregulated in both in vitro and in vivo atherosclerosis models [163]. It functionally interacts with hsa-miR-633 and enhances the proliferation, migration, and invasion of vascular smooth muscle cells (VSMCs), thereby promoting atherosclerosis progression. While previous studies have demonstrated the up-regulation of hsa_circ_0008896 in oxidized low-density lipoprotein-treated macrophages, there is limited research on its modulation in macrophages [164]. These findings suggest therapeutic potential of cell-specific ncRNAs in macrophages and emphasize the need for a deeper understanding of the interplay between macrophages and ncRNAs to expand therapeutic options for HF. Nevertheless, the translation of these therapeutic strategies to human studies has not been extensively realized. Successful clinical translation relies on a comprehensive understanding of the biology of target cells within specific organs and the specific non-coding RNAs involved, which can ensure the desired therapeutic outcomes while minimizing off-target effects.

### Biomarker study

Macrophages also secrete important signaling molecules that influence neighboring cells and the tissue microenvironment. Secreted factors from pathology-associated macrophages have the potential to be valuable biomarkers for the diagnosis and management of heart failure (Table 1).

#### Inflammatory mediators

##### Cytokines/chemokines

In response to various inflammatory stimuli, such as endotoxins and various forms of chemical and physical cardiac injury, activated macrophages primarily secrete pro-inflammatory cytokines. The elevated release of pro-inflammatory cytokines such as IL-1β, IL-6, MCP-1 and TNF-α intensifies pro-inflammatory signaling, ultimately leading to chronic inflammation and cardiac dysfunction in both HFrEF and HFpEF [79, 165, 166]. Notably, chronic and systemic inflammation, coupled with pro-fibrotic signals, holds greater prominence in HFpEF [167]. Elevated serum levels of pro-inflammatory macrophage cytokines, such as TNF-a, MCP-1, IL-6 and IL-12 were observed in patients with HFpEF compared to patients with asymptomatic left ventricular diastolic dysfunction or asymptomatic hypertension [80, 168]. Particularly noteworthy, a higher serum level of IL-6 is closely linked to the onset HFpEF in the general population, while no significant association with HFrEF [169]. While these cytokines may also originate from other damaged cardiac cells such as cardiomyocytes, endothelial cells, the reduction of pro-inflammatory signals in mice on a high-fat diet was notably achieved through the depletion of pro-inflammatory macrophages, resulting in mitigated diastolic dysfunction [170]. This finding strongly suggests that activated macrophages represent a primary cell source for the secretion of pro-inflammatory cytokines and underscores their potential as a promising therapeutic target in HFpEF.

##### MPO

Myeloperoxidase (MPO), an enzyme that facilitates the formation of hypochlorite from chloride and hydrogen peroxide, is another promising biomarker for the early detection of CVDs. It is released by activated macrophages and neutrophils during inflammatory processes [171, 172]. In the context of coronary artery disease (CAD), MPO contributes to oxidative stress and vascular dysfunction, both of which are key factors in the progression of the disease. Studies have shown that MPO levels are elevated in the blood and plaque tissue of patients with CAD, and that these levels correlate with disease severity [173]. However, MPO's specificity to CVD is not absolute, given that macrophage and neutrophil activation may also manifest in response to infections and non-cardiovascular-related inflammatory responses [172]. Despite these limitations, MPO is a valuable tool for the early detection of CAD. Its use in conjunction with other biomarkers and clinical risk factors can help to identify patients who are at high risk for developing the disease.

#### Pro-fibrotic mediators

##### Growth factors

Macrophages are also recognized for their pro-fibrotic properties in the advancement of heart failure. In particular, pro-fibrotic macrophages secrete various factors, including transforming growth factor (TGF)-β and IL-10, which orchestrate cardiac fibroblast activity-directing migration, proliferation, and collagen expression. This cascade ultimately leads to collagen fiber deposition and the formation of mature scars within the heart [73, 168, 174]. Correspondingly, HFpEF patients exhibit elevated plasma levels of TGF-β [175]. GDF-15, previously known as macrophage-inhibitory cytokine-1, belongs to the TGF-β superfamily and is another growth factor prominently secreted by activated macrophages [176]. While GDF-15 was initially identified as a negative regulator of macrophage activation, recent studies have revealed its paradoxical role in promoting fibrosis in various contexts. In the context of acute heart failure (AHF) patients participating in the RELAX-AHF study, elevated GDF-15 levels were correlated with a higher probability of adverse outcomes [177]. These findings suggest that GDF-15, like TGF-β, may contribute to the progression of heart failure. Although TGF-β and GDF-15 are not exclusive to cardiovascular disease, as they have also been found elevated in various cancers, their strong association with disease progression in heart failure patients suggests that these growth factors could serve as valuable tools for risk assessment and guiding therapeutic decisions.

##### Galectin-3

Galectin-3, a β-galactoside–binding lectin primarily secreted by activated macrophages, exhibits a strong association with myocardial fibrosis and the progression of HF [178–182]. Its involvement in cardiac fibrosis has prompted its inclusion in the European and American HF guidelines as a biomarker for myocardial fibrosis, with a class IIb recommendation [183, 184]. Gal-3 serves as a valuable biomarker not only for diagnosing HFpEF, but also for assessing post-MI risk and LV remodeling [185–188]. Multiple clinical studies have demonstrated that plasma levels of Gal-3 can effectively predict cardiovascular mortality [189, 190]. In particular, a serum Gal-3 level exceeding 8.7 ng/mL has been identified as an independent predictor of heightened all-cause mortality risk in both MI and chronic heart failure patients [191–193]. The strong association between Gal-3 levels and HF severity highlights its potential as a prognostic marker and therapeutic target. Measuring Gal-3 levels can aid in risk stratification and guide treatment decisions in HF patients.

#### Others: cell-to-cell networker

##### Exosomes

Exosomes, extracellular vesicles released by various cell types including macrophages, play a crucial role in intercellular communication via transferring a wide range of cargos, encompassing proteins or nucleic acids. Analyzing their presence in blood and their expression profile offer valuable insights into the function of macrophages in CVDs. In atherosclerosis, macrophage foam cells release a higher quantity of exosomes compared to normal macrophages. These additional vesicles play an important role in cell-to-cell crosstalk between macrophages and vascular smooth muscle cells (VSMCs) [194]. Exosomes originating from foam cells may stimulate VSMC adhesion and migration by modulating the actin cytoskeleton and focal adhesion pathways [194]. Moreover, under hypertensive conditions, the infiltration of macrophages significantly increases, leading to the upregulation of pro-inflammatory factors within their secreted exosomes, such as intracellular adhesion molecule-1 (ICAM1) and plasminogen activator inhibitor-1 (PAI-1). These exosomes further induce inflammation in endothelial cells, contributing to the progression of the pathological state [195].

##### Micro RNAs

MicroRNAs constitute a significant portion of the cargo within exosomes, and macrophage-derived miRNAs serve as potential biomarkers in CVDs. In the context of atherosclerosis, Zhang YG et al. demonstrated that macrophages involved in atherosclerotic processes release exosomal miR-146a. This miRNA exacerbates atherosclerosis progression by inducing neutrophil extracellular traps (NETs) and increasing oxidative stress in neutrophils. They also showed higher serum levels of miR-146a in atherosclerosis patients compared to healthy counterparts [196]. Interestingly, intracellular miR-146a and secreted miR-146a exhibited opposite effects on atherosclerosis progression. Intracellular miR-146a displayed anti-inflammatory properties in macrophages, leading to reduced atherosclerotic plaque formation [152, 153]. These contrasting effects may be attributed to differences in effector cells and cellular environments. In the context of MI, pro-inflammatory macrophages generate exosomal miR-155, which suppress angiogenesis in endothelial cells, consequently accelerating MI-associated injury [197]. Moreover, macrophage-derived miR-155 functions as a paracrine regulator, influencing fibroblast proliferation and inflammation. Mimicking miR-155 increases the risk of cardiac rupture by suppressing fibroblast proliferation and amplifying inflammation. Inhibiting miR-155 has demonstrated therapeutic potential, with improved cardiac function in a mouse model of MI [198]. Consistent with these findings, serum levels of miR-155 were significantly elevated in post-MI heart failure patients compared to both healthy individuals and MI patients without HF [199]. These findings highlight the potential of macrophage-derived miRNAs as biomarkers and therapeutic targets in CVDs.

#### Gene expression

Analyzing gene expression patterns offers valuable insights into macrophage function and their impact on HF progression, particularly following MI. Chen et al. conducted a transcriptomic analysis of peripheral blood mononuclear cells (PBMCs) from AMI patients (post-MI HF vs. post-MI non-HF) as well as performed single-cell RNA-seq (scRNA-seq) of recruited macrophages from a mouse model of MI (AMI vs. control mice). They further identified 25 common genes from both gene profiling datasets. These 25 genes were enriched in myeloid leukocyte activation, collagen metabolic process, and response to hypoxia, suggesting a close relationship with cardiac remodeling. Among the 25 identified genes, three genes emerged as promising biomarkers for early heart failure detection following acute MI: CUX1, CTSD, and ADD3. CUX1 and CTSD expression in cardiac macrophages exhibited a steady increase during the first week post-MI in a mouse model. Similarly, these two genes were upregulated in the peripheral blood of MI patients with heart failure compared to those without HF development. Conversely, ADD3 expression was significantly decreased in the HF context. Collectively, macrophage-associated genes hold promise as reliable biomarkers for cardiac remodeling and heart failure manifestation following MI. However, further extensive clinical investigations are warranted [200].

## Challenges and opportunity of macrophage-targeting strategies

### Heterogeneity: monocyte-derived macrophages vs resident macrophages

One of the challenges in macrophage-targeting therapies lies in the inherent heterogeneity and plasticity. Cardiac macrophages can be sub-divided into two populations by their origins: monocyte-derived macrophages from hematopoietic stem cells (HSCs) and embryonic yolk sac-oriented resident macrophages [69]. Current advances in transcriptomic analysis have enabled the clear differentiation of these two distinct subsets of macrophages in the heart. CCR2^−^ resident macrophages specifically express Lyve1, Cd163, Ccl24, while CCR2^+^ infiltrating macrophages are characterized by the expression of antigen-presenting genes (MHC II clusters), monocytes expressed gene (Ly6c2) genes [201]. Despite sharing common macrophage characteristics such as phagocytosis, immune response, and cytokine secretion, these two subsets exhibit divergent responses to injuries. Reiterating the points made earlier, monocyte-derived macrophages are primarily linked to inflammatory responses and pathogenic characteristics, while resident macrophages exhibit cardioprotective functions and play specific roles in cardiac physiology [69, 71, 104, 139, 165]. Consequently, depleting the entire macrophage population in the heart can lead to compromised LV remodeling, partially due to the loss of resident macrophages, which is crucial for the maintenance of myocardial homeostasis and cardiac function [202, 203]. Therefore, it is crucial to selectively target between bone marrow-derived macrophages and tissue-resident macrophages, while further discerning subsets and functions within the mixed population. Moreover, the inflammatory environment may shift the characteristics of cardiac macrophages from a reparative to a detrimental phenotype in the context of a diseased heart [201]. Therefore, the same intervention can have opposite outcomes depending on the timing and subset of macrophages targeted. To optimize therapeutic efficacy, it is imperative to comprehend the diverse macrophage types and their specific functions at the right timing to develop appropriate therapeutic approaches aimed at maintaining a balanced transition between protective and pathogenic responses in cardiac tissue.

### Targeted drug delivery to tissue macrophages

**Fig. 2:**
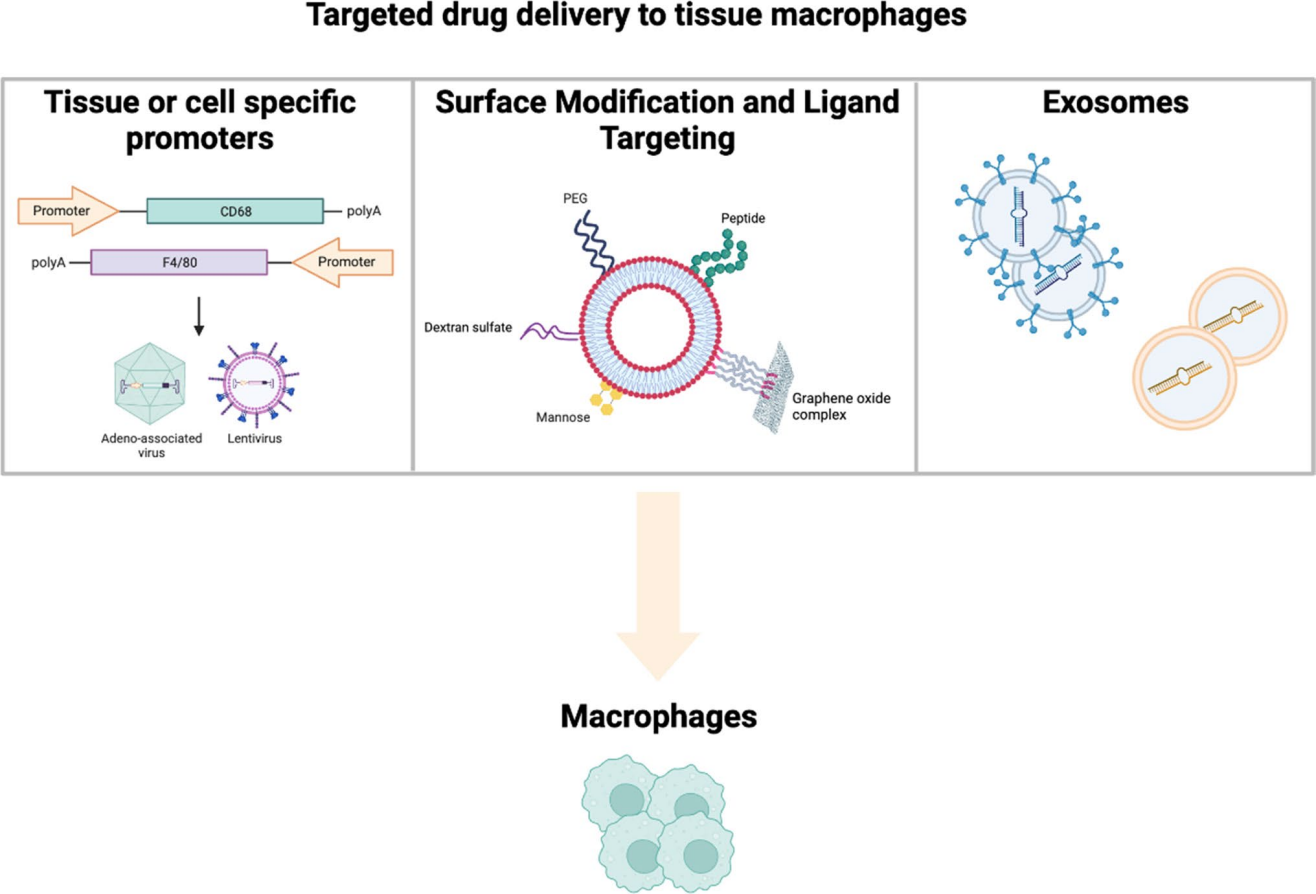
Targeted drug delivery to tissue macrophages. Effectively targeting cardiac macrophages remains a significant challenge in the field of cardiovascular medicine. With the development of advanced drug delivery strategies that precisely home in on macrophages, the promise of successful clinical translation can be realized. Several promising strategies are emerging, including tissue or cell-specific promoters, cell surface modification, exosome-based delivery *CD68* Cluster of Differentiation 68, *PolyA* polyadenylic acid, *PEG* polyethylene glycol

Despite of promising therapeutic potential of the strategies to target pathology-associated cardiac macrophages, to translate this promise to clinical application, challenges for the specific target delivery to tissue macrophages still remain to be overcome. Therefore, the development of advanced drug delivery strategies that precisely target macrophages is expected to bring the vision of successful clinical translation to reality. In this section, we will highlight the strengths and limitations of these strategies, with the ultimate goal of facilitating successful clinical translation (Fig. 2).

#### Utilize tissue or cell specific promoters

In the field of gene therapy, the utilization of tissue- or cell-specific promoters is a well-established method to ensure that the therapeutic effect is confined to the desired tissue cells. This technique has been widely applied in virus-mediated delivery systems, such as lentiviruses and adeno-associated viruses (AAV). Among these, the CD68 promoter stands out as one of the most commonly used promoters for directing transgene expression specifically to macrophages in in vivo settings via viral vectors. A study led by Levin et al. aimed to compare three variants of the CD68 promoter to determine the most efficient variant for macrophage-specific targeting using lentivirus. They observed that the 150 bp proximal region of the CD68 promoter outperformed the other variants and resulted in the highest macrophage specificity and significant protein expression, which could lead to maximal target efficiency [204]. Similarly, macrophage-specific promoters (F4/80 or CD68) also were applied to AAV vectors. Moreover, the cell specific transduction efficiency of recombinant AAV can be also enhanced by modification of capsids. Rosario et al. developed a recombinant AAV6 with triply mutated capsid Y731F/Y705F/T492V, under the control of macrophage-specific promoters (F4/80 or CD68), which allowed for selective gene modification of tissue macrophages while minimizing off-target effects [205]. This study was the first to use unique capsid/promoter combinations for tissue-macrophage-specific gene targeting, significantly enhancing safety and efficiency. AAV-mediated systems have been actively developed to deliver target molecules to the heart, and combining these systems with macrophage-specific promoters could boost target efficiency without altering other organ macrophages. However, since CD68 targets general macrophages regardless of whether they are resident or circulating, additional information for targeting pathological macrophages would also be beneficial for efficiency. Additionally, since viral vectors have a size limitation for cell uptake, combining them with nanoparticles is another emerging option to achieve specific targeting of cardiac macrophages with minimal side effects.

#### Surface modification and ligand targeting

As a result of challenges in delivering exogenous nucleic acids to macrophages via viral methods, several non-viral strategies have been explored. Nanoparticles and liposomes offer promising alternatives for delivery of small molecule such as siRNAs or miRNAs. These delivery vehicles can enhance cell binding and uptake specificity by incorporating ligands that specifically recognize cell surface receptors or markers expressed in target tissue cells. Recent advances in RNAi technology have enabled the selective targeting of desired cells by combining cell-specific targeting siRNAs with organ-specific biocompatible nanoparticles. For example, Tao et al. constructed PLGA-based siRNA nanoparticle conjugated with the peptide S2P, which binds to the scavenger receptor stabilin-2 on plaque macrophages. This nanoparticle was used to deliver siRNA targeting Ca2 + /calmodulin-dependent protein kinase γ (CamKIIγ), a plaque-destabilizing molecule, to atherosclerotic macrophages. In a mouse model of atherosclerosis, western diet-fed Ldlr^−/−^ mouse, intravenous injection of this nanoparticle significantly improved efferocytosis of macrophages and reduced atherosclerotic lesions [206]. Another example is the development of macrophage-targeting/polarizing graphene oxide (GO) complex (MGC) by Han et al. MGC was constructed by conjugating GO with polyethyleneimine (PEI) and folic acid-polyethylene glycol (FA-PEG), which has shown specific affinity for folate receptors (FR) on macrophages. In a mouse model of MI, intramyocardial injection of the MGC has delivered IL-4 pDNA selectively to cardiac macrophages, with limited uptake in cardiomyocytes and fibroblasts. From a therapeutic perspective, this delivery of IL-4 pDNA via MGC led to attenuated inflammation, thanks to the early shift of macrophages to a reparative phenotype and improved cardiac function [207]. An additional illustrative instance involves the high affinity between dextran sulfate (DXS) and the scavenger receptor class AI (SR-AI), which has been harnessed for drug delivery to macrophages. SR-AI plays a pivotal role in the recognition of modified lipoproteins and the recruitment of macrophages. Its specific ligand, DXS, has been shown to inhibit the cellular internalization of ox-LDL by blocking SR-AI. Based on this knowledge, Zhao and colleagues devised nanoparticles that target atherosclerotic macrophages by decorating their surfaces with DXS. Interestingly, when these nanoparticles were incubated with Apo-I, not only decrease the formation of macrophage-derived foam cells, but cholesterol efflux was also promoted [208]. Nevertheless, their therapeutic efficacy in an in vivo model of atherosclerosis remains to be demonstrated. Additionally, membrane surface glycoproteins, such as mannose, which are predominantly expressed on M2 (CD206^+^) macrophages, have also been investigated as targeting motifs for efficient drug delivery. Notably, mannosylated nanoparticles increase siRNA delivery to primary macrophages by fourfold compared to the same carrier without targeting moieties [209]. Despite the advancements in non-viral nanocarriers for gene delivery, certain challenges remain. Further research is needed to address issues such as suboptimal intracellular trafficking, low gene transfer efficiency, and the sustainable maintenance of stable gene expression.

#### Exosome-based delivery

Exosomes are extracellular vesicles (EVs), produced in the endosomal compartment, which mediate cell-to-cell communication by transferring biological material to neighboring cells. Taking advantage of this property, exosomes are coming to the light as a surrogate for drug delivery strategies. Exosomes carry diverse constituents including proteins, lipids, DNA, mRNA, miRNA, lncRNA, and circular RNA. Among these molecules, exosomal miRNAs are the most abundant type of exosomal cargo. Nguyen et al. reported the enrichment of miRNAs in atherogenic macrophage-secreted EVs from mouse and human. Among them, miR-146a is the most enriched miRNAs and EV-mediated transfer of miR-146a plays a pivotal role in the progression of atherosclerosis [210]. Likely, exosome contents from other cells can be also transferred to macrophages. This is the case of miR-182 containing exosomes released from mesenchymal stem cells (MSC) to macrophages, which are able to modulate the polarization of macrophages. In a mouse model of I/R injury, the intramyocardial injection of miR-182 enriched MSC-derived exosomes elicited the polarization of macrophages towards a reparative phenotype as well as improved the cardiac functional parameters. While these therapeutic effects have been significantly hindered when macrophages are depleted or miR-182 in MSC-exosomes is reduced, suggesting the exosomes can be applied for miRNA delivery to macrophages [148]. MSC-derived miR-101a also presented protective effects on cardiac remodeling post-MI via macrophage polarization [211]. Additionally, cardiosphere-derived cells (CDCs) derived exosomes or EVs also have been used as macrophage-targeting gene delivery cargos. In mouse and pig models of ischemic injury, the intramyocardial injection of miR-181b enriched CDC-exosomes favored the polarization of macrophages to M2 phenotype, which in turn alleviated the inflammatory and scar formation process [212]. Interestingly, exosomes can be structurally modified to enhance their delivery efficiency to cardiac tissue. For example, in the study conducted by Vandergriff et al., exosomes derived from cardiac stem cells were conjugated with a cardiac homing peptide (CHP) [213], suggesting macrophage-targeting conjugates may enhance the delivery specificity of exosomes to cardiac macrophages. While exosomes are emerging as delivery platform to target desired cells with their ability to be administered via various routes, limited toxicity, and structural stability, they still face critical challenges, including immunogenicity, a short half-life in circulation, limited target specificity, and a lack of standardized protocols for isolation, purification, and production. More importantly, for efficient macrophage targeting, nonspecific uptake and degradation of target molecules should be further considered.

## Ex vivo cell therapy (engineered cell therapy)

However, selective targeting of organ-specific macrophages, especially cardiac macrophages without influencing other cell types or organs is still challenging, due to the lack of studies of the unique marker for distinctive organ macrophages. Recent evidence has highlighted ex vivo reprogrammed macrophages could be an alternative option to avoid the massive in vivo delivery obstacles [214]. They can be re-educated to promote regeneration and reduce inflammation ex vivo. One of the key advantages of ex vivo reprogrammed macrophages is that they can be personalized to the individual patient. This is because they are derived from the patient's own cells, which reduces the risk of rejection. Additionally, ex vivo reprogramming allows for the creation of large numbers of macrophages with specific desired phenotypes, such as a reparative phenotype.

Ex vivo reprogrammed macrophages have been shown to be effective in a variety of preclinical models of cardiac repair. Bone marrow-derived macrophages (BMDMs) are a major cell source and are widely employed in macrophage-based cell therapeutic strategies for in vivo applications. While the multilineage potential of CD34^+^ cells may induce adverse remodeling by promoting inflammatory characteristics, hindering long-term improvements in cardiac function, direct transplantation of fully differentiated macrophages has emerged as an alternative cell therapy strategy. Notably, macrophage modification has substantially reduced the rejection risk of transplanted cells [215–217]. Transplantation of reparative macrophages, generated by re-educating BMDMs with the combination of M2 stimulators like IL-4, IL-10, and TGF-β1 [215, 216], has shown a superior therapeutic efficacy with improved cell engraftment in donor tissue compared to naïve BMDMs. Hypoxia priming is one of multiple strategies to educate macrophages toward reparative phenotype ex vivo. Transplantation of hypoxic macrophages primed with 1% oxygen immediately after the induction of MI in mice at 3 sites along the infarct border zone results in enhanced resolution of inflammation and subsequent better cardiac repair [218]. In line with this, Ben-Mordechai et al. have confirmed the crucial role of macrophages in the in vivo treatment of MI. The administration of mesenchymal stem cells (MSC) to mice following MI resulted in a greater polarization of macrophages towards an M2 phenotype within 3–4 days. While, temporary elimination of macrophages, with or without MSC treatment, was associated with a higher mortality rate due to left ventricular dysfunction and the development of excessive scarring. This effect was reverted by macrophage restoration and treatment with MSC, supporting the crucial role of these phagocytic cells in cardiac repair [219]. Moreover, the re-educated macrophages can function as adjuvant and positively influence regenerative stem cell therapies. In a rat model of MI, the therapeutic effects of the bone marrow-derived mesenchymal cells (BMMSCs) were strongly enhanced when they were co-injected with BMDMs compared to the only BMMSCs injected group. Injection of the mixture of BMMSCs and BMDMs notably improved cardiac function in terms of increased micro-vessel density as well as decreased fibrosis, and more importantly, facilitated resolution of inflammation with a higher presence of anti-inflammatory macrophages [220]. To date, the attempts to induce cardiac regeneration by stem cells have still not successfully translated to clinical studies, mainly due to the limited survival of the transplanted cells within a prolonged phase of tissue inflammation. These results suggest that macrophages can be an alternative adjuvant to overcome the challenges of current stem cell therapies including improved engraft survival and regulatory effects on inflammation.

Currently, several clinical trials employing ex vivo macrophage polarization and subsequent adoptive transfer for the treatment of cardiovascular diseases, such as ischemia, cardiomyopathy, or arterial diseases, have advanced to phase 2 or 3 [221]. In clinical trials, cell therapy mainly uses two sources of cells: autologous and allogeneic. Autologous transplants use the patient's own cells as the cell donor, while allogeneic transplants use cells from healthy donors. Autologous transplantation is a more traditional method and does not require the identification of an HLA-matched donor. Moreover, graft failure of autologous transplants is rare due to rapid immune reconstitution and a lower risk of life-threatening complications. Autologous re-educated macrophages have been an alternative source for cell transplantation in clinical studies. In a clinical pilot study of 21 children with severe cerebral palsy, autologous M2 macrophages significantly boost motor and cognitive activities of patients without adverse effects and comorbidities during 5-year follow-up [222]. Clinical efficacy of M2 macrophages also has been proved in patients with stroke (ClinicalTrials.gov, NCT01845350, completed). Intrathecal injection of autologous M2 macrophages improved neurological recovery with no serious adverse events or cell rejection for 6-month follow-up via immunomodulatory activity of M2 macrophages [223]. In contrast, a separate study by Perin et al. found that patients with chronic ischemic heart disease who were administered non-expanded autologous bone marrow macrophages via transendocardial injection did not experience any improvement in cardiac function compared to the placebo group (ClinicalTrials.gov, number NCT00824005, completed) [224], highlighting the *ex vivo* expansion of macrophages is crucial for a positive outcome in cardiac repair of patients with HF. In the cardiac field, an innovative patient-specific multicellular therapeutic approach called Ixmyelocel-T was established to treat patients with ischemic HF (ClinicalTrials.gov, NCT01670981, completed) [225, 226]. Ixmyelocel-T, an expanded multicellular therapy, is generated by expanding mixture of patient-derived autologous BMMCs enriched for CD45^+^CD14^+^M2 BMDMs and CD90^+^BMMSCs [225, 226]. In phase 2b, patients with dilated cardiomyopathy treated with transendocardial injection of Ixmyelocel-T showed an improvement in cardiac functions compared to patients treated with placebo during the 12 months following treatment administration [225, 226]. More importantly, the Ixmyelocel-T cells containing M2-reparative macrophages showed efficient clearance of apoptotic cells, which limit tissue injury and facilitate wound healing [227], indicating the therapeutic potential of reparative macrophages. Despite of their therapeutic potential, the development of Ixmyelocel-T as drug has been halted due to no further plan to initiate a phase III trial (Vericel Corporation, formerly Aastrom Biosciences). While the largest trial of Ixmyelocel-T, RESTORE-CLI clinical trial for limb ischemia has been downgraded in recent reports, due to the concerns about risk of bias, imprecision, and inconsistency (ClinicalTrials.gov, number NCT00468000, completed) [228]. Moreover, adverse events including amputations, heart attacks and strokes have been detected in patients with Ixmyelocel-T [228]. These clinical outcomes serve as a reminder that while cell therapy has the potential to revolutionize the treatment of CVDs, the safety and efficacy of new cell therapies must be carefully assessed before they are made widely available.

### Induced pluripotent stem cell (iPSC)-derived macrophages

However, standard autologous immune cell therapies have yet several limitations: (1) the transplanted cells are not able to effectively integrate into ischemic tissue; (2) autologous approaches using macrophages derived from patients who are often heavily pre-treated, which have hindrances in terms of manufacturing and efficacy (Table 2). Given these considerations, reprogramming of allogeneic macrophages, generated from healthy donors would strengthen the efficiency of macrophage-based cell therapy, iPSC-derived macrophages, therefore, have continued to improve and have endured as an effective treatment option against cardiac diseases. The therapeutic potential of human(h)iPSC-derived macrophages has been studied in different diseases. This is the case of pulmonary alveolar proteinosis, a pathological condition in which a mutation in the granulocyte–macrophage colony-stimulating factor (GM-CSF) receptor impedes the differentiation and function of alveolar macrophages. Happle et al. demonstrated that, in a murine model of pulmonary alveolar proteinosis, the intratracheal administration of hiPSC-derived macrophages results in efficient pulmonary engraftment, differentiation to tissue-specific alveolar macrophages and improvement of pulmonary functions [229]. Differently, in an immunodeficient mouse model of liver injury, Pouyanfard et al. demonstrated the beneficial effects of applying hiPSC-derived macrophages with an anti-inflammatory phenotype. In this case, hiPSC-derived macrophages were polarized towards an anti-inflammatory phenotype and injected intraperitoneally in mice with liver injury resulting in reduced fibrosis and inflammation [230].

**Table 2:** Autologous vs. Allogeneic macrophage cell therapy and their application

	Autologous	Allogenic
Cell source	Patient derived (same person)	Healthy donors
Immune compatibility	Lower risk of rejection No immunological tests required	Higher risk of rejection Immunological tests required
Cell characteristics	Diseased and/or heavily pretreated cells	Healthy cells
Practicability	Less efficient since cells need to be isolated and modulated Limited production (cells are from 1 person)	Cells are prepared and ready for direct application Expansion of production possible
Examples of application	Clinical studies: Ischemic cardiomyopathy [225] Cerebral palsy [222] Stroke [223]	Animal studies: Doxorubicin-induced heart failure in mice [215] MI treatment in mice [216] Human IPSC macrophages application in pulmonary alveolar proteinosis mice [229]

The beneficial effects of allogeneic macrophages have been also shown in cardiac disease model, rats subjected to MI. Here, human macrophages were isolated from the blood of donors and activated ex vivo by hypo-osmotic shock, which is a method to stimulate macrophages by transferring them into distilled water for a short time. This triggers cytokine production of macrophages and increases their phagocytosis efficiency [231]. Administration of the activated human macrophages into infarcted myocardium of rats leads to a significant improvement in heart function, tissue vascularization, and tissue repair within 5 weeks following treatment compared to the control group [232]. These favorable effects might be due to the replenishment of immune activity via the exchange of impaired old macrophages with healthy macrophages.

Chimeric antigen receptor (CAR) has become a promising approach to enhance the target recognition of immune cells. In a recent study on cancer research conducted by Klichinsky et al., macrophages were transduced by AAV to express CAR. The CAR-macrophages demonstrated a specific affinity and efficient antigen-specific phagocytosis for cancer cell targeting [233]. Emerging studies have addressed the importance of macrophage-mediated removal of apoptosis in cardiac remodeling post-injury. CAR-macrophage-targeting necrotic cardiomyocytes or activated fibroblasts could extend their therapeutic possibilities to cardiac conditions, in addition to cancers.

## Future outlook

### Advance macrophage study model in context of CVDs

**Fig. 3:**
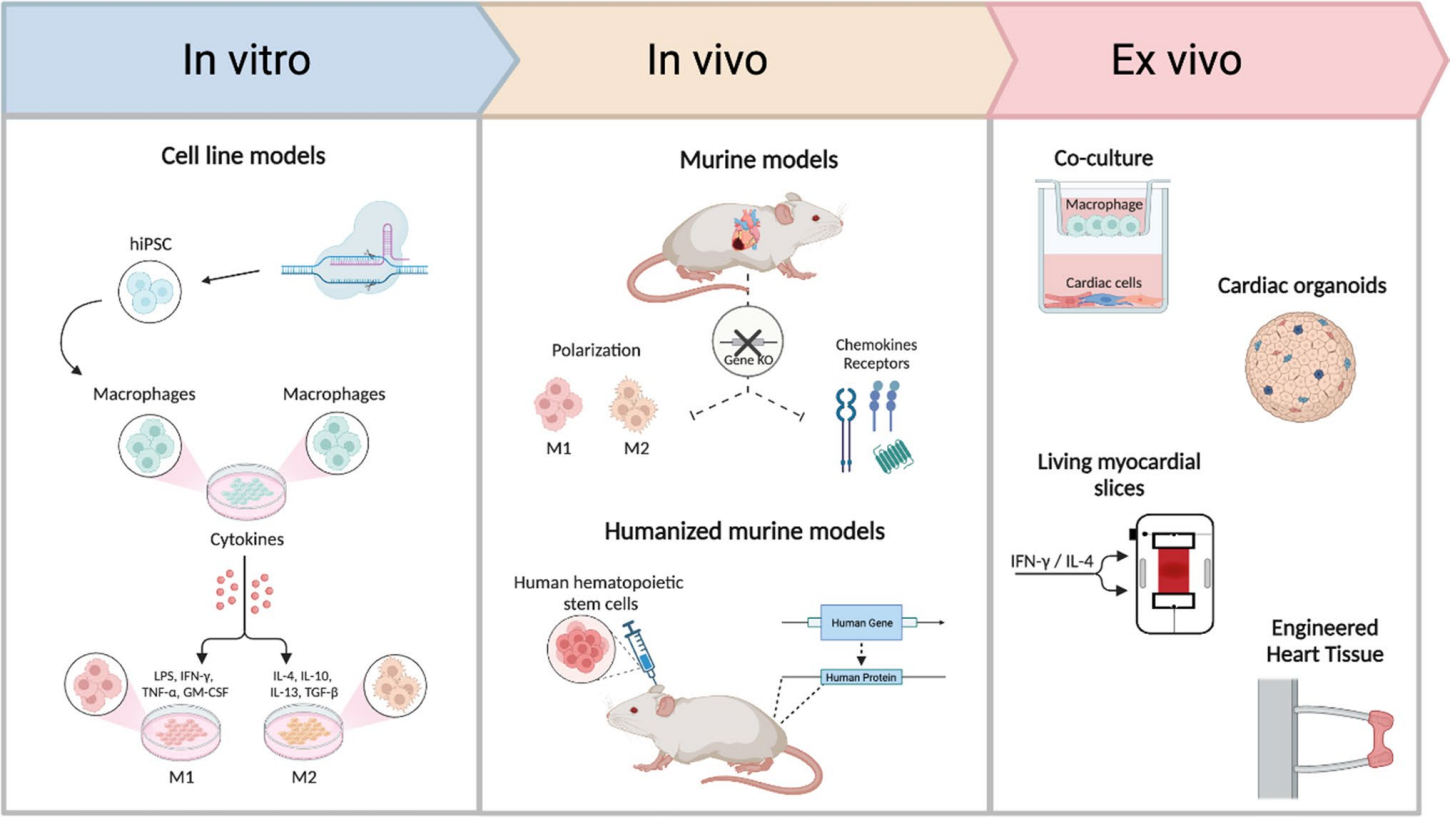
Advanced cardiac macrophage study model. For successful translation of research findings into clinical applications, more clinically relevant study models for cardiac macrophages, particularly in the context of cardiovascular diseases (CVDs), should be considered. We have proposed possible models ranging from in vitro to ex vivo settings using human induced pluripotent stem cell (iPSC)-derived macrophages or novel patient-derived heart slice models, living myocardial slices (LMS). *hiPSC* human pluripotent stem cells, *LPS* lipopolysaccharide, *IFN-γ* Interferon gamma, *TNF-α* tumour necrosis factor alpha, *GM-CSF* granulocyte–macrophage colony-stimulating factor, *IL* interleukin, *TGF-β* transforming growth factor-β, *KO* knock out

To facilitate the successful translation of therapeutic strategies targeting macrophages, it is imperative to establish appropriate frameworks for evaluating these modalities. In this regard, we provide a concise review of existing macrophage study models and propose a novel model to bridge the gap between in vitro and in vivo findings, ultimately advancing their clinical applicability in the context of CVDs (Fig. 3).

#### In vitro study

First and foremost, the current in vitro studies employing the classic M1/M2 macrophage paradigm are overly simplistic and fail to account for the heterogeneity and plasticity of macrophage physiology in response to pathological conditions. The traditional in vitro models involve inducing M1 macrophages through cytokines like LPS, IFN-γ, TNF-α, and GM-CSF, while M2 macrophages are activated using IL-4, IL-10, IL-13, and TGF-β [234]. This oversimplified model does not adequately represent the complexity of in vivo macrophage behavior. Recent advancements in high-throughput single-cell RNA sequencing have revealed diverse macrophage subsets associated with the progression of cardiovascular diseases, each with a distinct transcriptome [235]. Depending on the specific subsets of macrophages involved in different disease contexts, it is essential to use more precise stimulants and signaling pathways to generate the desired macrophage phenotype and function. Secondly, the choice of an appropriate cell model is crucial. For studying macrophage function, readily available immortalized cell lines such as murine Raw264.7 or human THP-1 cells are commonly used [236–238]. Despite their convenience and reproducibility, the findings from these cells often do not translate well into in vivo or clinical studies. This disconnect arises from the differences between these cell lines and tissue-resident macrophages [237–239].

While cardiac macrophages can be isolated from mouse or rat hearts and cultured ex vivo, there are challenges related to chronic culturing, limited cell yield, and disparities in genomic profiles when compared to humans. Consequently, human-induced pluripotent stem cell (hiPSC)-derived macrophages have emerged as an alternative in vitro model that better represents patient-derived pathology. Navarro-Guerrero and her colleagues have developed an efficient CRISPR/Cas9-knockout hiPSC-derived macrophage protocol through lentiviral transduction [240]. This approach has paved the way for the generation of clinically relevant human pathologic macrophages, which can be employed in drug screening. More importantly, exposure to organ-specific cues enabled human or murine iPSC-derived macrophages to differentiate into their respective tissue-resident macrophages. For example, murine iMacs, when injected into the brain, differentiated into microglia in vivo, while those engrafted in the lung developed into functional alveolar macrophages. These observations suggest that cardiac-specific cues can induce iMacs to differentiate into cardiac-resident macrophages, potentially providing a clinically relevant in vitro model of cardiac macrophages [241].

#### In vivo study

Animal models of CVDs have been comparably well-established and closely resemble clinical scenarios, encompassing conditions like MI, transverse aortic constriction (TAC) pressure overload, and high-fat diet-induced models. Consequently, these models have been instrumental in exploring the dynamic changes and heterogeneity of macrophage populations in various HF etiologies and disease progressions. Many studies involving gene functions during macrophage polarization or phagocytosis have employed animal models with gene knockouts. Several gene functions during macrophage polarization or phagocytosis have often been studied in animal model of knockout. For a successful knockout model, a deep understanding of the lineage-specific factors and markers within the target organ or cell is crucial. Variations in macrophage phenotypes and the corresponding markers are identified by various research groups. For example, CSF1 and its receptor CSF1R are well-known primary factors involved in general macrophage differentiation. Genetic manipulations targeting these factors have led to the generation of macrophage depletion models. The selective restriction of CSF1R through the genetic deletion of the fms-intronic regulatory element (Fire), a super-enhancer of CSF1R, has effectively suppressed the differentiation of tissue macrophages [242]. Additionally, mice with the CSF1R gene floxed (CSF1R^fl/fl^) have been widely used to ablate specific macrophage genes. Common phenotypic markers for macrophages include CD11b, F4/80, or CD68. Recent advances in imaging techniques, such as spatio-temporal single-cell RNA sequencing, have enabled the visualization and analysis of cardiac macrophage behavior and interactions within various in vivo CVD settings. While it is important to note that the immune milieu can be significantly influenced by the induction of specific pathologies. For example, different in vivo models of atherosclerosis, such as APOE-/- and LDLR-/- mice or high-fat diet-fed animals, may yield slightly different outcomes in terms of macrophage behavior and immune responses [243, 244]. Therefore, it's crucial to carefully select the appropriate model to ensure the accurate interpretation of research findings. Furthermore, many CVDs silently progress over extended periods, involving multiple etiologies and comorbidities [1]. To gain a more comprehensive understanding of macrophage roles and phenotypic shifts over time, long-term in vivo studies are necessary. Additionally, addressing the translation gap between small rodents and humans is vital [245, 246]. To bridge this gap, the use of large animal models or humanized mice is strongly recommended, as they better recapitulate the individual variations seen in clinical cases.

#### Ex vivo study

While in vivo studies provide valuable insights into diverse human CVDs, they still present several challenges, including cost, time, and ethical considerations. An emerging alternative experimental model to study cardiac macrophages is the ex vivo cell culture model, which serves as a compromise between in vitro and in vivo models. A protocol developed by Aktories et al. has enabled the establishment of long-term monocultures of tissue macrophages derived from distinct organs, offering a more clinically relevant ex vivo macrophage model [247]. Co-culturing ex vivo-isolated cardiac macrophages with other cardiac cells provides an indirect approach to understand their network dynamics from the acute to chronic phases of disease [248].

Furthermore, there has been significant progress in developing patient-derived heart or cell-based ex vivo culture platforms that mimic macrophage dynamics more closely to the clinical scenario. These platforms include precision heart cut slices, Engineered Heart Tissue (EHT), and hiPSC-derived organoids. Waleczek et al. employed the ex vivo culture model of precision heart slices, referred to as living myocardial slices (LMS), as a tool to investigate CRM. LMS are ultrathin multicellular cardiac preparations with the circulatory network interrupted, enabling the study of tissue-macrophage responses to immunomodulatory and mechanical stimulations within the preserved intricate myocardial architecture [248]. Importantly, LMS can also be generated from heart failure patients, allowing researchers to understand and visualize CVDs-associated behavior of cardiac macrophages, including their number, phenotype, and related signaling. Similarly, EHT has been introduced as another powerful ex vivo model for both healthy and diseased hearts. While EHTs are typically fabricated using cardiomyocytes, they better recapitulate the contractile function and complexity of the native myocardium with the addition of non-myocytes, such as fibroblasts and endothelial cells [249]. Given the emerging importance of resident macrophages in cardiac function, including electrical conduction and regeneration, the addition of macrophages in EHT is being strongly considered to create more physiologically relevant models [250]. In addition to LMS and EHT, a 3D culture model of mixed cell populations, iPSC-derived organoids, have emerged as promising tools for studying human cardiac cells [251–254]. Co-culture platforms containing human iPSC–derived cardiomyocytes and macrophages have demonstrated the intimate interactions between these two cell types [255, 256]. While these models are still in the early stages of development, the incorporation of damaged cardiomyocytes or fibrosis-associated fibroblasts into these systems holds promise as an experimental tool to study cardiac macrophages in the context of CVDs. Subsequent high-throughput transcriptomic or proteomic analyses of these settings could identify novel therapeutic targets for cardiac macrophages. However, to enhance clinical translation, further exploration of accurate toxicity assessment and experiment reproducibility is needed.

## Conclusion

In this review, we have outlined the latest advances in macrophage-based therapies for CVDs, from preclinical animal models to clinical trials, highlighting their potential for developing therapeutic interventions in the future. Over the past few decades, there has been tremendous growth in the development of macrophage-based therapeutic strategies, as these cells play a crucial role in diverse pathological conditions. In particular, the successful outcomes of immune cell therapies such as CAR-T cells have drawn attention to macrophages, the largest immune cell population. While macrophages are excellent phagocytes with homing properties and have shown great efficacy against cancers, their proportion and network are more complex in the heart, where they continuously change phenotype in response to environmental stimuli and closely interact with both diseased and healthy cells. Moreover, delivering drugs to cardiac macrophages remains challenging. These limitations make the use of therapeutic macrophages for treating CVDs more difficult. Therefore, novel interventions are required to improve selective targeting and durability without causing unspecific off-target effects for safe and long-lasting therapeutic effects. Precise and targeted modulation of macrophages holds potential for therapeutic interventions to prevent adverse cardiac remodeling, enhance tissue repair, and improve cardiac function in various CVDs. Our review has summarized the progress of macrophage-based therapeutic strategies to date and discussed additional ways to improve their efficacy, such as through the use of nanoparticles or cell transplantation. It is important to note that macrophage-targeted therapies are still in the early stages of development, and further research is needed to fully understand their efficacy and safety profiles in clinical settings. Although further research is needed to translate these basic findings into clinical treatments, these promising therapeutic strategies are paving the way for a new era of personalized medicine based on cellular treatments that could extend to a broader range of inflammatory pathologies.
